# Correlates of Health Satisfaction in a Web-Based Sample Enriched for Self-Reported Polycystic Ovary Syndrome: An Exploratory Cross-Sectional Study

**DOI:** 10.3390/healthcare14213635

**Published:** 2026-10-27

**Authors:** Elena Domnica Bacușcă, Alberto Emanuel Bacușcă, Agnes Iacinta Bacușcă, Elena Teona Coșovanu, Elena Popa, Antoneta Dacia Petroaie, Ana Maria Slănină, Mihaela Manole, Elena Adorata Coman

**Affiliations:** “Grigore T. Popa” University of Medicine and Pharmacy, 16 Universitatii Street, 700115 Iasi, Romania; elena-domnica.bacusca@email.umfiasi.ro (E.D.B.); agnes.bacusca@umfiasi.ro (A.I.B.); cosovanu_elena-teona@d.umfiasi.ro (E.T.C.); elena.popa@umfiasi.ro (E.P.); antoneta.petroaie@umfiasi.ro (A.D.P.); ana.slanina@umfiasi.ro (A.M.S.); mihaela.manole@umfiasi.ro (M.M.); elena.coman@umfiasi.ro (E.A.C.)

**Keywords:** polycystic ovary syndrome, polyendocrine metabolic ovarian syndrome, patient reported outcome measures, personal satisfaction, hirsutism, anxiety, body mass index, cross-sectional studies, health surveys

## Abstract

Background/Objectives: Polycystic ovary syndrome (PCOS), named polyendocrine metabolic ovarian syndrome by a 2026 global consensus, combines metabolic, dermatological and psychological features. We examined which clinical, anthropometric and symptom-related characteristics were associated with health satisfaction in a web-based sample enriched for self-reported PCOS. Methods: Cross-sectional web-based survey using a Romanian-language questionnaire (October–November 2023). Of 212 submissions retained after integrity screening, 159 (75.0%) reported a previous diagnosis of PCOS. Health satisfaction (study-specific five-point item) was analysed with proportional-odds ordinal regression including seven covariates, irrespective of diagnostic status (*n* = 194). Sensitivity analyses restricted the model to respondents reporting a diagnosis, adjusted for diagnostic status or sociodemographic characteristics and retained all submissions. Results: Lower health satisfaction was associated with higher body mass index (OR 0.92 per kg/m^2^; 95% CI 0.87–0.97), self-reported hirsutism (OR 0.43; 95% CI 0.24–0.76) and reported anxiety (OR 0.49; 95% CI 0.28–0.87; reference group mostly “do not know/no answer”). Estimates changed little after these adjustments or with all submissions; among respondents reporting a diagnosis (*n* = 145), the estimates for self-reported hirsutism and reported anxiety were attenuated and imprecise. Age, menstrual irregularity, infertility diagnosis and dysmenorrhea severity showed no statistically detectable association. Conclusions: These exploratory, mutually adjusted associations involve higher body mass index, self-reported hirsutism and endorsement of the questionnaire’s anxiety option; the last reflects a response contrast, not clinically assessed anxiety. They warrant confirmation in clinically characterised cohorts using validated instruments.

## 1. Introduction

Polycystic ovary syndrome (PCOS) is among the most common endocrine disorders in women of reproductive age, and pooled prevalence estimates vary substantially with the diagnostic criteria applied and the population studied [1]. Diagnosis rests on two of three Rotterdam features (clinical or biochemical hyperandrogenism, ovulatory dysfunction and polycystic ovarian morphology) after exclusion of conditions with an overlapping presentation [2]. In May 2026 a multistep global consensus process adopted polyendocrine metabolic ovarian syndrome (PMOS) as the new name for the condition, on the grounds that the former name placed inaccurate emphasis on ovarian cysts; its implementation, including a transition period and alignment with disease classification, is under way [3]. Because the data were collected in 2023, when the questionnaire used the term PCOS, the historical term is retained throughout.

The disorder is not confined to the ovary. Hyperandrogenism and insulin resistance with compensatory hyperinsulinaemia reinforce one another and, together with neuroendocrine and adipose-tissue dysfunction, generate reproductive, metabolic and dermatological features [2,4,5]. This endocrine-metabolic core underlies the elevated long-term risks of metabolic syndrome and type 2 diabetes that accompany the condition [4,6]. Hirsutism, acne and androgenetic alopecia, the cutaneous manifestations of androgen excess, are frequent and visible [2]. Psychological comorbidity is prominent: affected women have substantially higher odds of moderate-to-severe depressive and anxiety symptoms than unaffected women, independently of obesity [7].

Each of these domains has been linked to reduced health-related quality of life (HRQoL) measured with validated instruments. Affected women score lower across physical and, most consistently, emotional dimensions of generic questionnaires [8], while disease-specific measures identify hirsutism and menstrual dysfunction as the most affected areas [9]. The instruments used include the 36-item Short Form Survey, the Polycystic Ovary Syndrome Questionnaire (PCOSQ) and its modified version [10,11]. Which symptom domains weigh most heavily on perceived health is less settled. Reported determinants of quality of life include body mass index (BMI) and age [12], while hirsutism has been identified as one of the most affected disease-specific domains [9]. Psychological symptoms are also common in PCOS [7]. The relative contribution of these clinical and psychological features varies across populations and measurement instruments. Recent web-based surveys using validated instruments have linked obesity and negative body image to poorer HRQoL and mental health [13], and hirsutism, acne and oligomenorrhea to lower body appreciation [14].

Patient-reported evidence from Central and Eastern Europe remains limited. The studies we identified used heterogeneous instruments, ranging from disease-specific measures to questionnaires developed for the individual study, and relied on convenience or single-centre recruitment [12,15,16]. Adiposity-related, hyperandrogenic and psychological features are also interrelated: anxiety symptoms in PCOS have been associated with higher BMI and hirsutism scores [7], and obesity with negative body image and anxiety symptoms [13,14]. In the studies cited, however, psychological symptoms were analysed as outcomes of physical features [7,13,14] or as a domain of HRQoL [8,9], rather than jointly with adiposity and hyperandrogenic features as mutually adjusted correlates of an overall appraisal of health. Among the studies we identified, none examined correlates of health satisfaction in a web-based sample enriched for women reporting a previous diagnosis of PCOS; the Romanian patient-reported data we located address sexual function and body image in a clinic-based sample of 54 women [17]. Web-based recruitment offers efficient access to a frequently under-diagnosed and geographically dispersed population and has been applied to this question elsewhere in the region [15].

The principal objective was to identify which clinical, anthropometric and symptom-related characteristics were associated with health satisfaction among women participating in this web-based survey. Secondary objectives were to describe patient-reported symptom burden, to examine overall quality-of-life satisfaction in a parallel model and to assess whether the associations persisted among respondents reporting a diagnosis of PCOS and after accounting for diagnostic status. Respondents reporting and not reporting a diagnosis were also compared descriptively.

## 2. Materials and Methods

### 2.1. Study Design and Reporting

This was an exploratory, cross-sectional, web-based observational study using a Romanian-language online questionnaire. Data were collected through an online questionnaire open from 10 October to 17 November 2023, which was also available as a PDF on request (Section 2.2). Reporting follows the Strengthening the Reporting of Observational Studies in Epidemiology (STROBE) statement for cross-sectional studies [18]; the completed checklist is provided as Table S5. Some items of the Checklist for Reporting Results of Internet E-Surveys (CHERRIES) [19] were not applicable because the recruitment denominator and the number of individuals who viewed the invitation were not available; therefore, view and participation rates could not be calculated. Platform-level duplicate-response prevention settings were not documented; the post hoc data-integrity screen applied to the export is described in Section 2.6.

### 2.2. Participants and Recruitment

Eligible participants were adult women (≥18 years) who voluntarily completed the questionnaire. Recruitment used non-probability convenience sampling. The Romanian-language questionnaire was administered using Microsoft Forms (Microsoft Corporation, Redmond, WA, USA). The survey link was distributed privately through patient contacts associated with medical practices and related groups. An identical PDF version was available by e-mail upon request. Of the 219 questionnaire records, 177 were completed directly online and 42 using the PDF version. The completed PDF questionnaires were subsequently entered into the same electronic questionnaire system by one author, and the transcribed responses were independently checked against the original PDF forms by a second author. The consolidated data export therefore contained all 219 records. The invitation, reproduced in File S1, described a study of the symptomatology of polycystic ovary syndrome and its impact on quality of life and stated that participation was voluntary and that responses would be treated confidentially. The resulting sample was enriched for women reporting a previous diagnosis of PCOS, who accounted for 159 of the 212 submissions retained after the integrity screen (Section 3.1), consistent with this dissemination context, although participation was not restricted to women with a diagnosis. The sample was not intended to estimate prevalence or to provide population-representative estimates.

Study size was determined by the number of eligible respondents who completed the questionnaire during the predefined recruitment period. No a priori sample-size calculation was performed; the precision of each estimate is conveyed by its 95% confidence interval.

### 2.3. Questionnaire and Variables

The questionnaire was a structured, self-administered instrument assembled for this survey, comprising items on sociodemographic characteristics, anthropometry, menstrual and reproductive history, dermatological features of hyperandrogenism, psycho-emotional status and comorbidities. The clinical and patient-reported items were intended to capture the principal clinical features of PCOS and the associated symptom burden as perceived by respondents. Several clinical terms were accompanied by brief lay explanations in parentheses to aid comprehension (for example, hormone measurements were explained as blood tests for hormones). The nine patient-reported items used five-point response scales, seven with extent anchors and two (negative feelings and physical pain) with frequency anchors. The wording of every item used in the analyses, in the original Romanian with an English translation prepared by the authors, is given in Supplementary File S1. The questionnaire was administered only in Romanian; the English translations were prepared for reporting and were not used for data collection.

The questionnaire included study-specific single-item ratings of satisfaction with overall quality of life and with health, developed with reference to WHOQOL concepts [20]. The health item corresponds conceptually to the WHOQOL-BREF item on satisfaction with health, and the quality-of-life item to its item on overall quality of life, which requests a rating rather than a satisfaction judgement; neither reproduces the WHOQOL-BREF wording or response anchors. Both study items asked to what extent the respondent was satisfied, on a five-point scale from “not at all” to “to a very great extent”. As in the corresponding WHOQOL-BREF item, the health item asked for a global appraisal of the respondent’s own health rather than a rating of specific symptoms or functions. Neither item specified a reference period, and both were phrased in the present tense (File S1). The complete WHOQOL-BREF was not administered. These two ratings and the remaining symptom and psycho-emotional items were written for this survey and had not been validated or pretested. No formal comprehensibility assessment, such as cognitive interviewing, was undertaken; the questionnaire was intended for exploratory description of patient-reported characteristics rather than for psychometric measurement.

Hirsutism was self-reported in response to the question “Do you have hirsutism (marked hairiness, excess hair on the chest, face, buttocks)?”, answered yes, no or “do not know/no answer”. The psychological-condition item (“Do you suffer from any psychological condition?”) was multi-response, with the options anxiety, depression, fear of infertility and “do not know/no answer”, and did not include an explicit “no” option. Respondents selecting anxiety were coded as reporting anxiety; those not selecting anxiety were coded as not reporting anxiety. The resulting variable is termed reported anxiety throughout; it reflects self-report on this item and does not denote a clinically diagnosed anxiety disorder or a score on a validated anxiety instrument. Its reference category comprises all respondents who did not select anxiety, including those who selected “do not know/no answer”, and therefore cannot be interpreted as respondents without anxiety. Depression and fear of infertility were coded in the same way. The response patterns for this item are reported in Table S10, and alternative codings were examined in sensitivity analyses (Section 2.7). Diagnosed obesity and other chronic conditions were derived from a multi-response question on chronic conditions diagnosed in the respondent. The multi-response treatment item was worded for respondents with a diagnosis of PCOS; treatment was therefore summarised and modelled for respondents reporting a diagnosis (Section 2.7; Table S12).

Because no multidimensional instrument was administered, the constructs are described as health satisfaction, overall quality-of-life satisfaction and patient-reported symptom burden rather than as health-related quality of life.

### 2.4. Classification by Self-Reported Diagnostic Status

Diagnostic status was derived from participants’ self-report of a previous diagnosis of PCOS. The questionnaire did not include a separate yes/no question on diagnosis. Classification used a single questionnaire item with conditional wording, which asked respondents with such a diagnosis which features had prompted them to seek medical advice: “If you have been diagnosed with PCOS (polycystic ovary syndrome), what prompted you to see a doctor?” The response options were irregular cycles, hirsutism, acne, weight gain, infertility, severe pain, heavy menstrual flow, other and the explicit alternative “I am not diagnosed with PCOS”; the original Romanian wording, response options and coding are given in File S1. The condition was expressed only in the item wording, and no other item recorded diagnostic status. The item permitted multiple selections, and no respondent combined the explicit alternative with a reason for seeking care. Of the 219 submissions in the platform export, respondents selecting that alternative were classified as reporting no diagnosis (*n* = 41), those leaving the item blank as undetermined (*n* = 13), and all remaining respondents, who selected one or more of the other options, as reporting a diagnosis (*n* = 165); the corresponding numbers after the integrity screen are reported in Section 3.1. Because the item wording addressed respondents with a diagnosis, selecting any reason for seeking care was taken as a report of a previous diagnosis; the particular reasons selected and the symptoms reported elsewhere in the questionnaire were not used to classify or reclassify any respondent, and the Rotterdam criteria were not applied retrospectively. The questionnaire did not record who had made the previous diagnosis or which diagnostic criteria had been used. Classification depended only on the recorded answers to this item, not on treatment responses or on any assumed routing between items. The treatment question was also conditionally worded, but responses to it were recorded for 18 of the 40 respondents in the primary analytical dataset who reported no diagnosis; these responses are described in the note to Table S12 and did not affect classification or the diagnosis-restricted models.

Diagnostic status was therefore not clinically confirmed by the Rotterdam criteria, biochemical testing or pelvic ultrasonography; self-report was used because clinical confirmation was not feasible in an online survey. Participants with undetermined status were excluded from comparisons by diagnostic status but remained eligible for the principal model, which did not use diagnostic status. The influence of diagnostic status on that model was examined in sensitivity analyses (Section 2.7).

### 2.5. Outcomes

Health satisfaction was the principal outcome. The analyses were not based on a prospectively registered analysis plan. Overall quality-of-life satisfaction was examined in a parallel model with the same covariate set; the remaining patient-reported items were secondary and descriptive.

### 2.6. Data Management and Derived Variables

Age was calculated from date of birth at the survey close date of 17 November 2023 and was treated as missing for five records with an implausible date of birth. All respondents with a valid date of birth were aged 18 years or older (range 18.5–45.6 years). For the five records with an implausible date of birth, adult age could not be confirmed from the recorded data; these records were retained in the descriptive analyses but, lacking age, were excluded from all regression models. Heights entered in metres, including those written with a decimal comma, were converted to centimetres, and unit suffixes were removed from anthropometric entries before analysis. BMI was calculated as weight (kg) divided by height (m) squared and classified by World Health Organization categories (underweight < 18.5, normal 18.5–24.9, overweight 25.0–29.9, obese ≥ 30.0 kg/m^2^); implausible weights (>200 or <30 kg) were set to missing. For waist circumference, the numeric component was extracted where a value was reported; blank entries, dashes and “do not know” responses were treated as missing. No exact duplicate records were found. A post hoc data-integrity screen identified seven potential repeat-submission pairs, which shared an identical date of birth, height and weight and gave largely concordant responses to the other items. The later submission in each pair was excluded from the primary analytical dataset (212 of 219 submissions retained), and all submissions were retained in a sensitivity analysis.

For single-choice items offering an explicit “do not know/prefer not to answer” alternative, that response was treated as missing rather than recoded as absence of the feature. This accounts for the differing denominators in Table 1. Reported gravidity and number of children were collected as free text and coded numerically. Multi-response items were recoded as binary indicators over all respondents, so an unticked option was coded as absent and these variables have no missing values. For the psychological-condition item, the response “do not know/no answer” was therefore coded as not reporting the condition. Responses to the five-point items were scored 1–5. Valid numbers and missing values for every variable are reported in Table S1. Height, weight and waist circumference were self-reported and self-measured without a standardised protocol.

The sociodemographic and treatment items used in the sensitivity analyses defined after peer review (Section 2.7) were coded as follows (File S1). Education, residential environment and marital status were answered by all respondents; lower and upper secondary education were combined because only two respondents reported lower secondary education, and divorced respondents (*n* = 3) were combined with single respondents. Occupation, recorded as free text, was coded into student or pupil, health professional, other employed or self-employed, and not in paid employment; entries that could not be classified (four respondents in the primary analytical dataset) were treated as missing. Each treatment option was coded as a binary indicator, and any treatment was defined as selection of any option other than “no treatment”. One respondent selected “no treatment” together with metformin and myo-inositol; this response was coded as treated, and alternative handling was examined.

### 2.7. Statistical Analysis

Continuous variables are summarised as median [interquartile range] and categorical variables as counts and percentages with their denominators. Associations between continuous measures were quantified with Spearman rank correlation (ρ) among all respondents in the primary analytical dataset with complete pairs, with 95% confidence intervals from 2000 bootstrap resamples.

The principal analysis was a proportional-odds ordinal logistic regression for health satisfaction. Age, BMI, menstrual irregularity, self-reported hirsutism, infertility diagnosis, dysmenorrhea severity and reported anxiety were entered simultaneously. The covariates were selected on clinical grounds to represent the demographic, adiposity, reproductive, hyperandrogenic, pain-related and psychological domains of clinical relevance in PCOS, each of which has been linked in previous studies to quality of life or psychological well-being in affected women [7,8,9,12,13,14]. Because these features are interrelated [7,13,14], entering them simultaneously allowed the association of each with health satisfaction to be examined with adjustment for the others within a common multivariable framework. The choice of indicators also reflected data availability: adiposity was represented by BMI, available for 211 of the 212 respondents, rather than by waist circumference, which was missing for 51 (Table S1). Treatment was not included in the pooled model because the item was conditionally worded for respondents reporting a diagnosis. Sociodemographic characteristics other than age, which have also been associated with quality of life [12], were not part of this clinically focused covariate set (Section 4.6). The covariate set followed the principal objective, which concerned clinical, anthropometric and symptom-related characteristics; the influence of sociodemographic characteristics, and that of treatment among respondents reporting a diagnosis, was examined in sensitivity analyses defined after peer review (see below). No automated variable-selection procedure was used, and the model was intended to describe mutually adjusted associations, not to estimate causal effects or to predict health satisfaction. The model was fitted by complete-case analysis on all respondents with complete covariate data, irrespective of diagnostic status (194 of the 212 submissions in the primary analytical dataset). Diagnostic status was not used to define the principal-model population because it was not an eligibility criterion and the principal question concerned correlates of health satisfaction across the recruited sample. Restriction to respondents reporting a diagnosis and adjustment for reported diagnostic status were therefore examined as sensitivity analyses. The proportional-odds assumption was assessed with the Brant test [21], and collinearity with variance inflation factors. Model fit was summarised using the likelihood-ratio test, McFadden and Nagelkerke pseudo-R^2^ and the Akaike information criterion (AIC). Unadjusted estimates were obtained from univariable proportional-odds models, and BMI was also expressed per 5 kg/m^2^. The functional form of age was examined in one robustness analysis that added a quadratic term for mean-centred age, compared with the linear model by a likelihood-ratio test. A second robustness analysis excluded the later of one additional pair of highly similar submissions that did not meet the matching rule of the integrity screen (Table S8). A parallel model for overall quality-of-life satisfaction, using the same covariate set and analysis population, is reported in Table S4.

In sensitivity analyses, the principal model was first restricted to respondents reporting a diagnosis of PCOS. It was then refitted among respondents with classifiable diagnostic status, with self-reported diagnostic status (no reported diagnosis versus reported diagnosis) added as a covariate, and among all submissions, including the seven later submissions excluded by the integrity screen. The proportional-odds assumption was assessed in each model.

Further sensitivity and specification analyses were defined after peer review and were therefore not prespecified; each retained the seven principal covariates, and the principal model was not modified (Table S13). First, reported anxiety was recoded by treating “do not know/no answer” on the psychological-condition item as missing, so that respondents reporting anxiety were compared with those selecting depression or fear of infertility without anxiety; the item was also entered as three categories (anxiety; depression or fear of infertility without anxiety; “do not know/no answer” only, as reference). Second, education and residential environment were added as indicators of socioeconomic context, which has been associated with quality of life in women with PCOS [12], followed by marital status. Occupation was examined separately among respondents with classifiable occupation; health professionals and other employed respondents were combined because several free-text entries could not be assigned unambiguously to either group.

Third, among respondents reporting a diagnosis who answered the treatment item, an indicator of any treatment, or alternatively of any listed medicine (oral contraceptive, metformin, spironolactone or flutamide, versus all other responses), was added, and the estimates were compared with those of the model without treatment fitted to the same respondents; the response selecting both “no treatment” and treatments was also coded as untreated or excluded. Treatment estimates were not interpreted as treatment effects. Fourth, a quadratic term for mean-centred BMI and, separately, a natural cubic spline for BMI with three degrees of freedom were compared with the linear term; dysmenorrhea severity was entered as categories, with moderate severity as reference, so that equal spacing between severity levels was not assumed; and, because the lowest health-satisfaction category was sparse, the two lowest categories were combined. Nested models were compared by likelihood-ratio tests, and the proportional-odds assumption was assessed with the Brant test where it was evaluable, that is, where none of its binary logistic submodels showed separation and the covariance matrix used by the test was positive definite.

Comparisons by self-reported diagnostic status were secondary and descriptive, restricted to the 199 respondents in the primary analytical dataset classifiable by that status. Continuous and ordinal variables were compared with the Mann–Whitney U test, with differences summarised as Hodges–Lehmann estimates and 95% confidence intervals. Binary characteristics were compared with Fisher’s exact test throughout, with differences summarised as conditional maximum-likelihood odds ratios and exact 95% confidence intervals. These comparisons are reported in Tables S2 and S3 and summarised in Section 3.5. With 40 respondents reporting no diagnosis, their precision was limited.

No adjustment for multiplicity was applied, consistent with the exploratory aim. Associations from the models are reported as odds ratios with 95% confidence intervals and are interpreted by the magnitude and precision of those estimates rather than by threshold alone [22].

**Figure 1:**
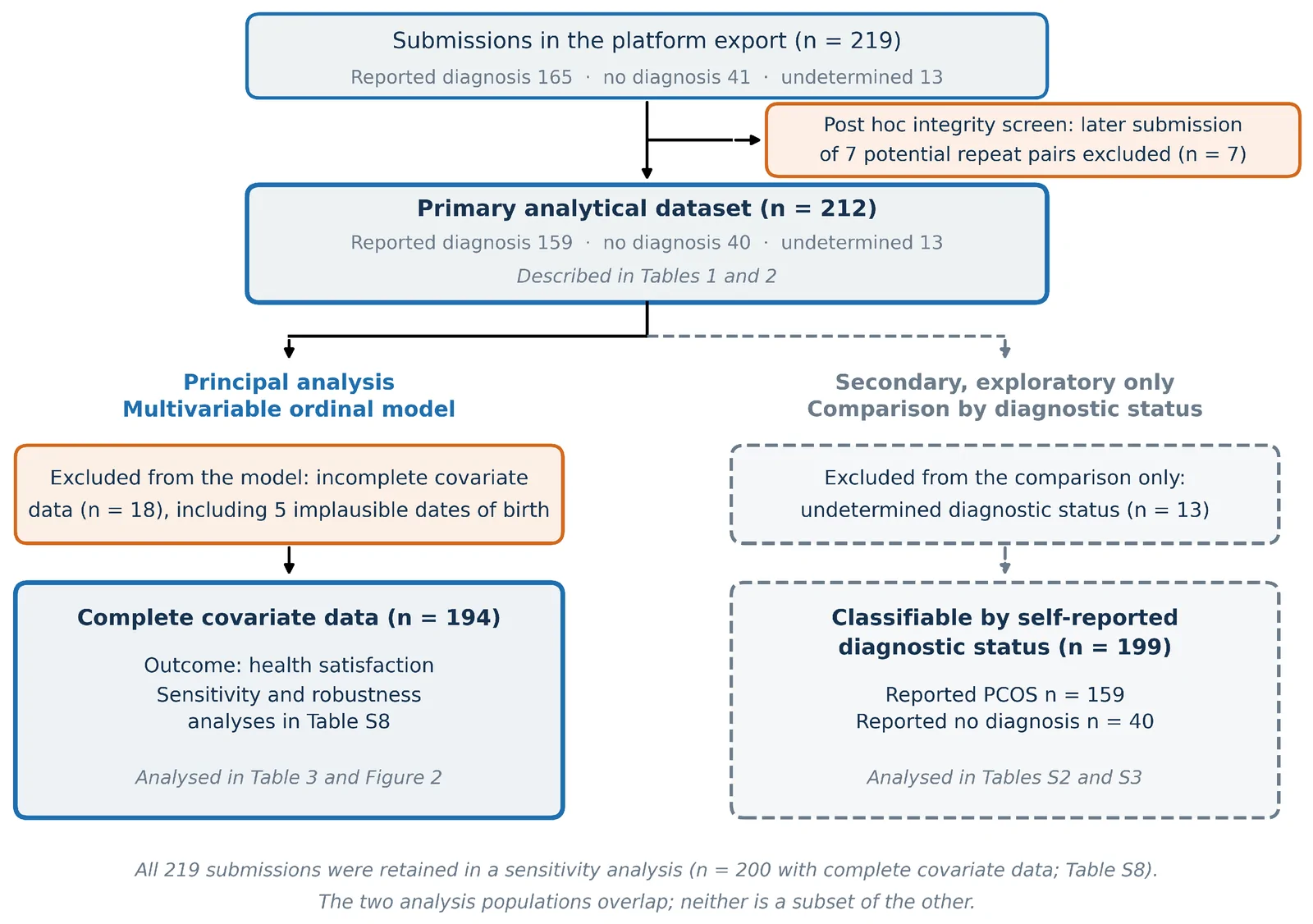
Flow of submissions through the study. A post hoc integrity screen excluded the later submission of each of seven potential repeat-submission pairs, leaving 212 submissions in the primary analytical dataset, which Table 1 and Table 2 describe. The principal ordinal model of health satisfaction (Table 3) was fitted on all respondents with complete covariate data, irrespective of diagnostic status (*n* = 194: 145 reporting a diagnosis, 37 reporting none and 12 undetermined). The secondary, exploratory comparisons by self-reported diagnostic status (Tables S2 and S3; dashed boxes) excluded 13 respondents with undetermined status, leaving 199 classifiable respondents. All submissions were retained in a sensitivity analysis (Table S8).

Table 1 and Table 2 describe the primary analytical dataset. The principal model and the diagnostic-status comparisons drew on overlapping subsets of it, derived from the original 219 submissions, and each table identifies its analysis population (Figure 1). A two-tailed *p* < 0.05 was treated as the conventional threshold. Analyses were performed in R version 4.3.3 (R Foundation for Statistical Computing, Vienna, Austria) with the packages readxl, MASS, brant and splines, starting from the original survey export.

**Table 3:** Factors associated with health satisfaction: principal multivariable proportional-odds ordinal logistic regression (*n* = 194).

Variable	OR	95% CI	*p*-Value
Age (per year)	0.98	0.93–1.03	0.403
BMI (per kg/m^2^)	0.92	0.87–0.97	0.001
Irregular cycle	1.05	0.60–1.84	0.876
Self-reported hirsutism	0.43	0.24–0.76	0.004
Infertility diagnosis	1.08	0.56–2.08	0.815
Dysmenorrhea (per level)	0.88	0.67–1.14	0.335
Reported anxiety	0.49	0.28–0.87	0.014

Proportional-odds ordinal logistic regression for health satisfaction (higher score = better); an OR below 1 indicates lower health satisfaction. Covariates were entered simultaneously; hirsutism was self-reported, and anxiety was reported on a multi-response item without an explicit “no” option; its reference category consisted mainly of respondents selecting “do not know/no answer” (Section 3.1). The model was fitted by complete-case analysis on respondents with complete covariate data (*n* = 194 of the 212 submissions in the primary analytical dataset), irrespective of self-reported diagnostic status. Diagnostic status was not entered as a covariate; sensitivity analyses are reported in Tables S8 and S13. BMI, body mass index; CI, confidence interval; OR, odds ratio.

### 2.8. Ethical Considerations

The study was conducted in accordance with the Declaration of Helsinki and approved by the Research Ethics Committee of the “Grigore T. Popa” University of Medicine and Pharmacy, Iași, Romania (approval no. 347, 28 August 2023). All participants provided informed consent before completing the questionnaire: online respondents acknowledged an introductory consent page, and consent for the PDF version was documented on the consent form. Date of birth was used to derive age and, together with height and weight, to screen for potential repeat submissions (Section 2.6). Both steps preceded statistical analysis, and direct identifiers and date of birth were not retained in the analytical dataset. Data were processed in compliance with the EU General Data Protection Regulation.

## 3. Results

### 3.1. Participant Characteristics

The platform export contained 219 submissions: 165 (75.3%) reported a previous diagnosis of PCOS, 41 (18.7%) reported none and 13 (5.9%) left the diagnostic item blank. The integrity screen excluded the later submission of each of seven potential repeat-submission pairs, leaving 212 submissions: 159 (75.0%) reporting a diagnosis, 40 (18.9%) reporting none and 13 (6.1%) undetermined (Figure 1). The principal model included the 194 respondents with complete covariate data: 145 reporting a diagnosis, 37 reporting none and 12 with undetermined status (Table S6). The 18 excluded respondents lacked data on self-reported hirsutism (*n* = 7), infertility diagnosis (*n* = 6), age (*n* = 5, implausible date of birth), menstrual regularity (*n* = 2) or BMI (*n* = 1); 2 lacked more than one covariate (Table S1). The comparisons by diagnostic status included the 199 classifiable respondents.

Table 1 summarises the 212 respondents in the primary analytical dataset, irrespective of diagnostic status. The sample was young, with a median age of 28.0 years [IQR 24.6–33.5] and a median BMI of 23.4 kg/m^2^ [20.5–27.4]. Overall, 74 of 211 respondents with BMI data (35.1%) had a BMI of 25.0 kg/m^2^ or more. Menstrual irregularity was reported by 122 of 210 respondents with data on that item (58.1%) and self-reported hirsutism by 100 of 205 (48.8%). Acne was reported by 117 of 211 (55.5%) and oily skin or seborrhea by 142 of 212 (67.0%). An infertility diagnosis was reported by 57 of 206 (27.7%) and prior hormonal testing by 164 of 209 (78.5%). Reported anxiety was recorded for 79 of 212 respondents (37.3%) and reported depression for 25 of 212 (11.8%). On the psychological-condition item, 116 respondents (54.7%) selected only “do not know/no answer”, an option never combined with another; 49 (23.1%) selected anxiety alone, 30 (14.2%) anxiety with depression or fear of infertility and 17 (8.0%) depression or fear of infertility without anxiety (Table S10). The reference category for reported anxiety therefore consisted mainly of respondents selecting “do not know/no answer” (116 of 133 in the primary analytical dataset and 110 of 125 in the principal-model population).

Valid numbers for every variable, including the continuous measures, are given in Table S1. Most respondents had completed higher education (188 of 212, 88.7%) and lived in an urban area (179 of 212, 84.4%); sociodemographic characteristics and the treatment reported by respondents with a diagnosis are given in Tables S11 and S12.

### 3.2. Patient-Reported Health Satisfaction and Symptom Burden

The median health satisfaction score was 3.0 [IQR 3.0–4.0] and the median overall quality-of-life satisfaction score was 4.0 [3.0–4.0] (Table 2). Among the symptom-burden items, premenstrual burden had the highest median (4.0 [3.0–4.0]) and pain interference with activities of daily living the lowest (2.0 [2.0–3.0]). Appearance was rated as important (median 4.0 [4.0–5.0]). Health-satisfaction responses were concentrated in the middle and upper-middle categories, selected by 63 (29.7%) and 91 (42.9%) of the 212 respondents, whereas the lowest and highest categories were selected by 9 (4.2%) and 11 (5.2%); the principal-model population showed the same pattern, with 7 of 194 (3.6%) and 10 of 194 (5.2%) in the extreme categories (Table S9). There was no floor or ceiling concentration, but the extreme categories were sparse.

### 3.3. Factors Associated with Health Satisfaction

**Figure 2:**
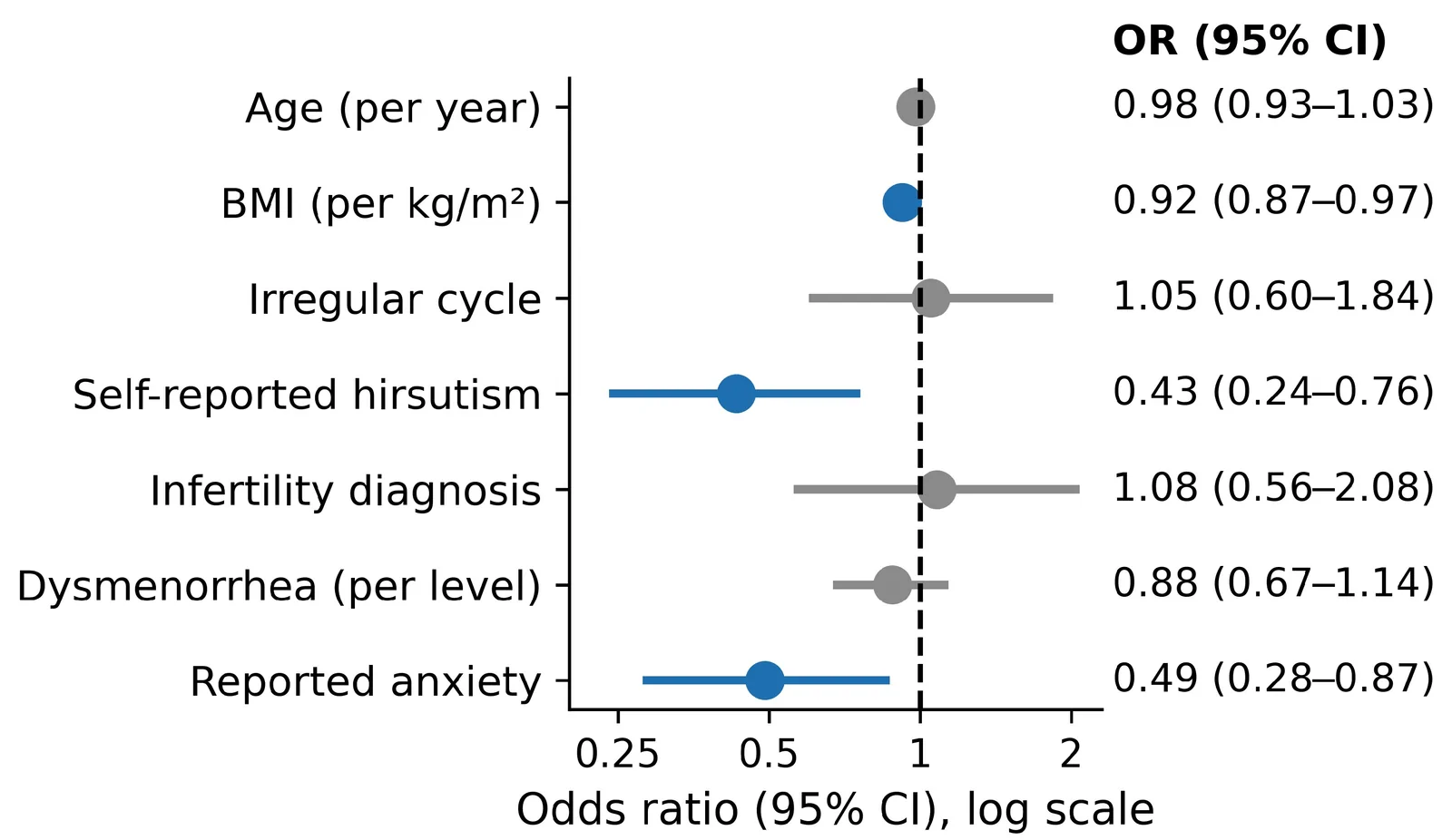
Forest plot of the principal multivariable proportional-odds ordinal logistic regression model for health satisfaction (*n* = 194). Points are odds ratios and horizontal lines are 95% confidence intervals; the dashed line marks the null value (OR = 1). Blue markers denote variables whose 95% confidence interval excludes 1 and grey markers those whose interval includes 1; the odds ratio and confidence interval are printed beside every row, so the figure can be read without relying on colour. For this outcome (higher score = better), an OR below 1 indicates lower health satisfaction. The reference category for reported anxiety consisted mainly of respondents selecting “do not know/no answer” (Section 3.1). BMI, body mass index; CI, confidence interval; OR, odds ratio.

In the principal proportional-odds ordinal model (*n* = 194), lower health satisfaction was associated with higher BMI (OR 0.92 per kg/m^2^; 95% CI 0.87–0.97; *p* = 0.001) (Table 3, Figure 2). It was also associated with self-reported hirsutism (OR 0.43; 95% CI 0.24–0.76; *p* = 0.004) and reported anxiety (OR 0.49; 95% CI 0.28–0.87; *p* = 0.014). Per 5 kg/m^2^, the OR for BMI was 0.64 (95% CI 0.49–0.84). Age, menstrual irregularity, infertility diagnosis and dysmenorrhea severity showed no statistically detectable association, and their confidence intervals included 1. Unadjusted estimates are given in Table S7.

The model was significant overall (likelihood-ratio χ^2^ = 36.19, df = 7, *p* < 0.001), with a McFadden pseudo-R^2^ of 0.07, a Nagelkerke pseudo-R^2^ of 0.18 and an AIC of 490.0. The Brant test showed no evidence against the proportional-odds assumption (χ^2^ = 21.38, df = 21, *p* = 0.44; all covariate-specific *p* > 0.10). Variance inflation factors ranged from 1.05 to 1.15, indicating no material collinearity. Allowing a nonlinear association with age did not materially alter the estimates for BMI, self-reported hirsutism or reported anxiety (quadratic age term: likelihood-ratio χ^2^ = 3.74, df = 1, *p* = 0.053; Table S8). Excluding the later of one additional pair of highly similar submissions (*n* = 193) also left these estimates essentially unchanged (Table S8). These pseudo-R^2^ values indicate modest model fit and are not literal proportions of outcome variation explained.

Unadjusted rank correlations in the primary analytical dataset were concordant in direction with the model. Health satisfaction correlated inversely with BMI (ρ = −0.30; 95% CI −0.42 to −0.17; *n* = 211) and with waist circumference (ρ = −0.35; 95% CI −0.48 to −0.22; *n* = 161). Overall quality-of-life satisfaction showed weaker inverse correlations with the same measures (ρ = −0.18; 95% CI −0.31 to −0.05; *n* = 211, and ρ = −0.23; 95% CI −0.36 to −0.08; *n* = 161).

### 3.4. Sensitivity Analyses

Among the 145 respondents reporting a diagnosis of PCOS, all three principal estimates kept their direction. The BMI estimate was similar (OR 0.93 per kg/m^2^; 95% CI 0.88–0.98), whereas the estimates for self-reported hirsutism (OR 0.55; 95% CI 0.29–1.05) and reported anxiety (OR 0.68; 95% CI 0.35–1.31) were attenuated and less precise, and their confidence intervals included 1. In this subgroup, the estimate for dysmenorrhea severity was 0.73 per level (95% CI 0.54–0.99; Table S8); given the number of estimates examined, this isolated result is not interpreted. Among the 182 classifiable respondents, the model adjusted for diagnostic status gave estimates close to the principal ones for BMI (OR 0.91; 95% CI 0.86–0.96), self-reported hirsutism (OR 0.45; 95% CI 0.25–0.82) and reported anxiety (OR 0.53; 95% CI 0.30–0.96); the diagnostic-status estimate itself was imprecise (OR 1.00; 95% CI 0.48–2.06). Retaining all submissions (*n* = 200) gave similar estimates for BMI (OR 0.91; 95% CI 0.87–0.96), self-reported hirsutism (OR 0.42; 95% CI 0.24–0.75) and reported anxiety (OR 0.46; 95% CI 0.26–0.81). The Brant test showed no evidence against the proportional-odds assumption in any of these models (omnibus *p* = 0.48, 0.49 and 0.57; Table S8).

In the analyses defined after peer review (Table S13), treating “do not know/no answer” as missing restricted the model to 84 respondents and changed the comparison: 69 respondents endorsing anxiety were compared with 15 endorsing depression and/or fear of infertility without anxiety. The estimate for reported anxiety was imprecise (OR 0.68; 95% CI 0.22–2.11), and because the comparator differed from that of the principal model, this analysis can neither validate nor invalidate the principal response contrast. In this smaller and differently composed population, the estimate for self-reported hirsutism was also attenuated and imprecise (OR 0.91; 95% CI 0.37–2.26). With the item entered as three categories (*n* = 194), the OR for reported anxiety relative to respondents selecting only “do not know/no answer” was 0.47 (95% CI 0.26–0.84); this analysis largely restates the principal contrast rather than independently confirming it.

Adding education and residential environment left the estimates for BMI (OR 0.92; 95% CI 0.87–0.97), self-reported hirsutism (OR 0.46; 95% CI 0.26–0.84) and reported anxiety (OR 0.48; 95% CI 0.27–0.85) close to those of the principal model, as did further adding marital status. Among the 191 respondents with classifiable occupation, adding occupation did not change the estimates; the lower estimate for self-reported hirsutism in this population (OR 0.38 with and without occupation) reflected the change in population rather than adjustment. The occupation estimates themselves were imprecise, particularly for the five respondents not in paid employment (OR 0.70; 95% CI 0.12–4.20). Among the 144 respondents reporting a diagnosis who answered the treatment item, 66 (45.8%) reported any treatment (Table S12). Adding this indicator left the estimates essentially unchanged relative to the model without treatment in the same respondents (BMI, OR 0.93 in both models; self-reported hirsutism, 0.53 versus 0.54; reported anxiety, 0.68 versus 0.69), and the treatment estimate itself was imprecise (OR 1.08; 95% CI 0.57–2.04). Alternative treatment codings gave similar results (Table S13).

Neither a quadratic term nor a natural cubic spline for BMI materially improved fit (likelihood-ratio *p* = 0.111 and 0.212), and the linear term was retained, although these tests have limited power and do not establish linearity. Entering dysmenorrhea severity as categories did not improve fit (*p* = 0.438), and the per-level term was retained; it assumes a constant change in the log-odds between adjacent severity levels, not clinically equal distances between them. Combining the two lowest health-satisfaction categories also gave similar estimates. All fitted ordinal models in Table S13 converged with finite standard errors. The Brant test, which relies on separate binary logistic submodels, showed no evidence against the proportional-odds assumption where it was evaluable (omnibus *p* = 0.21–0.68). It was not evaluable for the models treating “do not know/no answer” as missing, adding occupation or entering dysmenorrhea severity as categories, because sparse cells produced separation in these submodels, and it could not be computed for the spline model.

### 3.5. Exploratory Comparisons by Self-Reported Diagnostic Status

Respondents reporting a previous diagnosis differed from those reporting none in the direction expected for the condition, with greater waist circumference and more frequent menstrual irregularity, hirsutism, infertility diagnosis and prior hormonal testing (Table S2). This pattern is consistent with the expected clinical profile but does not independently validate the reported diagnostic classification, because several of these features were also represented among the reasons respondents could indicate for seeking medical care; prior hormonal testing may additionally reflect healthcare contact rather than phenotype.

No statistically detectable between-group difference was observed across the patient-reported items; effect estimates with 95% confidence intervals are given in Table S3.

### 3.6. Secondary Analysis of Overall Quality-of-Life Satisfaction

In the parallel proportional-odds model for overall quality-of-life satisfaction (*n* = 194), higher BMI was associated with lower satisfaction (OR 0.93 per kg/m^2^; 95% CI 0.89–0.99; *p* = 0.013). Estimates for the remaining covariates, including a nominal association with infertility diagnosis, were imprecise and are reported in Table S4; as this was a secondary model, they were not interpreted further. The parallel model yielded a Nagelkerke pseudo-R^2^ of 0.09, a descriptive index of model fit that does not represent the proportion of outcome variation explained.

## 4. Discussion

### 4.1. Principal Findings

In this web-based sample, three-quarters of respondents reported a previous diagnosis of PCOS, and in the principal proportional-odds model (*n* = 194) three characteristics were associated with lower health satisfaction after simultaneous adjustment: higher BMI (OR 0.92 per kg/m^2^; 95% CI 0.87–0.97), self-reported hirsutism (OR 0.43; 95% CI 0.24–0.76) and reported anxiety (OR 0.49; 95% CI 0.28–0.87). The first two are adiposity-related and dermatological characteristics. The third denotes endorsement of the questionnaire’s anxiety option, not clinically assessed anxiety or a validated psychological-domain score. Each estimate was somewhat attenuated relative to its unadjusted value but was not fully accounted for by the other covariates (Table S7). These estimates, from analyses that were neither prespecified nor adjusted for multiplicity, describe exploratory associations within the recruited sample rather than causal or disease-specific effects. This mutually adjusted evaluation complements studies that related these features to individual HRQoL domains or examined psychological symptoms as outcomes [7,9,13,14].

Adjustment for diagnostic status or sociodemographic characteristics, and retention of all submissions, left the three estimates close to the principal ones, and adjustment for treatment changed the estimates little among respondents reporting a diagnosis (Section 3.4; Tables S8 and S13). These analyses do not exclude residual confounding. Among respondents reporting a diagnosis, the BMI estimate was similar to the principal one, whereas the estimates for self-reported hirsutism and reported anxiety were attenuated and imprecise; these two associations are therefore less secure in that subgroup. The estimate for reported anxiety also depends most on the coding of its item, because its reference category consisted mainly of respondents selecting “do not know/no answer”. Excluding these respondents changed the comparison to respondents endorsing depression and/or fear of infertility without anxiety and gave an imprecise estimate, so this analysis can neither validate nor invalidate the principal contrast. The three-category analysis largely restates that contrast rather than independently confirming it. Age, menstrual irregularity, infertility diagnosis and dysmenorrhea severity showed no statistically detectable association.

The cross-sectional design precludes establishing the temporal direction of the observed associations, and unmeasured factors may contribute to them.

### 4.2. Comparison with Previous Literature

The association with adiposity agrees with studies identifying weight status as a correlate of reduced health-related quality of life in this population [13,23]. In a recent web-based survey of 587 women with PCOS, obesity and negative body image were jointly associated with poorer HRQoL and mental health [13]. A systematic review of randomised controlled trials found that weight-loss interventions improved insulin resistance, free androgen index and menstrual frequency, but did not significantly improve hirsutism or quality of life [24]. A cross-sectional association of this kind cannot show whether weight change alters perceived health.

The association with hirsutism matches a study of 796 women in which hirsutism showed the strongest association with quality of life, ahead of BMI, menstrual irregularity and infertility [23]. Meta-analytic work likewise identified hirsutism and menstrual dysfunction as the most affected domains [9]. In a recent Dutch web-based study, hirsutism and acne, together with oligomenorrhea, were associated with lower body appreciation [14]. Anxiety and depressive symptoms are common in women with PCOS [7], and reported anxiety was frequent in this sample (37.3%); because of the coding of the item, however, its association with health satisfaction is the least certain of the three (Section 4.1). The Androgen Excess–PCOS Society recommends that affected women be screened for depressive and anxiety symptoms at diagnosis [25]. A comparative study across Rotterdam phenotypes reported no statistically detectable difference in quality of life, anxiety or depression between phenotypes, although burdens relating to hirsutism, acne and menstruation did vary [26]. Because all participants in that study had the condition, it bears on the weight carried by individual features rather than by the diagnostic label.

These comparisons involve different instruments. The two global outcomes used here are study-specific single-item satisfaction ratings that resemble, but do not reproduce, the WHOQOL-BREF general items [20]. Single-item global ratings of health predict mortality in general populations [27] but do not provide domain-level measurement, and neither the full WHOQOL-BREF nor a disease-specific instrument such as the PCOSQ [10,28] or the PCOSQOL [29] was administered. Our results therefore agree in direction with the domain scores reported above but are not directly comparable with them.

### 4.3. Interpretation

Two of the three associated characteristics are visible, whereas the third, reported anxiety, reflects the endorsement of a questionnaire option. Adiposity and hirsutism are features a woman sees and that others may see. Women with PCOS rate their hirsutism as more severe than clinicians do, and their own ratings relate more closely to quality of life than clinician ratings [30]. Self-perceived severity may therefore be the construct most relevant to self-appraised health.

Menstrual irregularity, infertility diagnosis and dysmenorrhea severity showed no statistically detectable association with the outcome in the principal model. The sample was relatively young (median age 28.0 years; Table 1), and most respondents with available data reported having no children (168 of 205, 82.0%). These characteristics may have limited the opportunity for infertility to be investigated or recognised and restricted the variability of infertility diagnosis and other reproductive characteristics, which could have reduced the precision of the corresponding estimates. A single global rating of health may also be less sensitive than condition-specific instruments to cyclical or episodic symptoms such as dysmenorrhea. The absence of statistically detectable associations therefore does not indicate that menstrual irregularity, infertility or dysmenorrhea are unrelated to perceived health in the wider population of women with PCOS.

Unmeasured or incompletely measured factors, including treatment, may also contribute to the associations observed (Section 4.6).

### 4.4. Implications for Care

The associated characteristics correspond to weight-related and dermatological concerns and to self-reported anxiety, which can be considered concurrently in patient-centred clinical assessment. The data do not show that modifying any of them would improve health satisfaction, and the anxiety finding does not reflect clinically assessed anxiety.

The domains identified correspond to those addressed by existing international guidance, which recommends endocrine and metabolic care, dermatological treatment of hyperandrogenic features, structured lifestyle support and routine mental-health assessment [2]. Cognitive behavioural therapy may reduce depressive symptoms in affected women, although the pooled estimate is imprecise [31]. A multi-methods study combining an online survey with interviews described women feeling dismissed and inadequately informed during the diagnostic encounter [32]. Clinical recommendations rest on this guideline and intervention evidence rather than on cross-sectional survey data.

In the Romanian context, these data come from a Romanian-language, web-based convenience sample enriched for self-reported PCOS, in which most respondents were young, lived in urban areas and had completed higher education (Section 4.6); they therefore do not describe Romanian women with PCOS as a whole. In this sample, adiposity-related and dermatological characteristics and endorsement of the questionnaire’s anxiety option were each associated with health satisfaction after mutual adjustment, so the characteristics associated with respondents’ appraisal of their health spanned several areas rather than one. For clinical practice in Romania, this pattern points to the value of enquiring about weight-related and cutaneous concerns together during PCOS consultations, in keeping with the emphasis of the international guideline on improved models of care, shared decision-making and adaptation to regional health systems [2]. Because anxiety was not clinically assessed here, attention to psychological concerns in such consultations rests on existing recommendations to screen for anxiety and depressive symptoms [2,25] rather than on these data. Such combined enquiry is a clinically reasonable approach to assessment rather than an intervention evaluated in this study; its effect on health satisfaction, and the applicability of these associations to women with clinically confirmed PCOS, remain to be examined in clinically characterised Romanian cohorts.

### 4.5. Exploratory Comparisons by Diagnostic Status

The comparisons by self-reported diagnostic status showed no statistically detectable between-group difference across the patient-reported items; these exploratory comparisons were not designed to estimate a disease-specific effect. The group reporting no diagnosis comprised 40 participants from the same web-based sample, and among them 32.5% reported irregular cycles, 28.9% hirsutism and 7.7% an infertility diagnosis. Unrecognised or unreported PCOS in this group is therefore plausible, and such misclassification could attenuate a true contrast. The small size of this group also limits precision (Tables S2 and S3), so the absence of detectable differences does not imply equivalence. The effect of adding diagnostic status to the principal model is reported in Section 3.4.

### 4.6. Strengths and Limitations

The study contributes patient-reported data collected using a Romanian-language web-based questionnaire; regional evidence has come mainly from Poland and Serbia [12,15,16] and, in Romania, from a clinic-based study of sexual function [17]. The principal model was checked for the proportional-odds assumption and collinearity and was examined in sensitivity and specification analyses (Tables S8 and S13), and denominators and missing values are reported for every variable.

The study has several limitations. Recruitment used non-probability online convenience sampling, a recognised source of selection bias [33], and the sample was enriched for respondents reporting a previous diagnosis of the condition. The sample does not support prevalence estimates, and generalisability beyond similarly recruited samples is limited. Most respondents were young, lived in urban areas (179 of 212, 84.4%) and had completed higher education (188 of 212, 88.7%; Table S11). Women reached through an electronic invitation circulated via contacts associated with medical practices may also differ from other women with PCOS in healthcare contact, recognition of symptoms and willingness to report them, and the associations observed may therefore not apply to women who are older, live in rural areas or have less formal education. View, participation and completion rates could not be computed, and duplicate-response prevention settings were not documented. A post hoc integrity screen identified seven potential repeat-submission pairs; the later submission in each pair was excluded from the primary analysis, and an analysis retaining all submissions yielded materially similar estimates. Diagnostic status was based on participants’ report of a previous diagnosis, derived from an item on the reasons for seeking care, and was not confirmed by the Rotterdam criteria, hormonal testing or ultrasonography [34]. Some diagnostic misclassification is possible, given the recognised heterogeneity of diagnostic criteria and clinical phenotypes [35]. Because classification relied on an item with conditional wording rather than a direct question, respondents who misread the item, or whose previous diagnosis had not been established by recognised criteria, may also have been misclassified. Such misclassification bears most directly on the analyses that used diagnostic status, but it also affects how far the sample was enriched for PCOS and hence the population to which the findings apply. Because the principal model pooled respondents irrespective of diagnostic status, it describes associations within the recruited sample and does not establish associations specific to PCOS. No exclusion was applied for pregnancy, lactation, menopause or for the conditions the Rotterdam criteria require to be ruled out, and time since diagnosis was not recorded.

The two global outcomes were study-specific single-item ratings, and no multidimensional quality-of-life instrument was administered. These ratings and the other symptom and psycho-emotional items were written for this survey without formal validation or pretesting, and their wording did not specify a reference period. Reported anxiety and self-reported hirsutism were each assessed with a single item rather than with the GAD-7, the HADS or a modified Ferriman–Gallwey score. Self-assessment also tends to rate hirsutism as more severe than clinicians do [30]. The psychological-condition item did not include an explicit “no” option, and respondents selecting “do not know/no answer” were coded as not reporting anxiety; they made up 110 of the 125 respondents in the reference category of the principal model. The estimate for reported anxiety therefore contrasts respondents endorsing anxiety mainly with respondents who did not give a definite answer. Excluding the latter changed the comparator to 15 respondents endorsing depression and/or fear of infertility without anxiety and gave an imprecise estimate, which can neither validate nor invalidate this contrast. This reference group may include respondents with unreported anxiety as well as respondents without it, and the direction of any bias arising from this coding cannot be determined from these data. Because exposures and outcome were self-reported at one time point, shared-method variance is possible. Anthropometric measures were self-reported.

The treatment item was worded for respondents with a diagnosis and could not be analysed for the whole sample. Among respondents reporting a diagnosis, adjustment for any treatment changed the estimates little, but the item did not record dose, duration, timing or indication, and confounding by treatment or by indication cannot be excluded. Education, residential environment, marital status and occupation were not part of the principal model; adding them in sensitivity analyses changed the estimates little, which does not exclude residual confounding by these or other characteristics. Income and smoking status were not collected. The model was fitted by complete-case analysis on 194 of the 212 submissions in the primary analytical dataset, and listwise deletion reduces precision and can introduce bias when data are not missing at random [36,37]. Finally, the cross-sectional analyses, including those defined after peer review, were exploratory and not prespecified, and no adjustment was made for multiplicity.

## 5. Conclusions

In this Romanian-language, web-based sample enriched for self-reported PCOS, higher body mass index, self-reported hirsutism and reported anxiety were associated with lower health satisfaction in an exploratory multivariable ordinal model. The principal estimates were similar in models adjusted for diagnostic status or sociodemographic characteristics, although the estimates for self-reported hirsutism and reported anxiety were attenuated and imprecise among respondents reporting a diagnosis; the association with reported anxiety also rests on a reference group composed mainly of respondents who gave no definite answer. The model did not provide evidence of associations with age, menstrual irregularity, infertility diagnosis or dysmenorrhea severity.

These findings identify adiposity-related and dermatological characteristics, together with endorsement of the questionnaire’s anxiety option, that were each associated with patient-reported health satisfaction after mutual adjustment. They do not establish an association with clinically assessed anxiety or with a validated psychological-domain score, and they warrant confirmation in clinically characterised cohorts. Such studies should use validated multidimensional patient-reported outcome measures and validated anxiety instruments, record treatment for all participants and follow a prospectively specified analysis plan.

## Figures and Tables

**Table 1:** Sociodemographic, anthropometric and clinical characteristics of the primary analytical dataset (*n* = 212).

Characteristic	Value
Age, years	28.0 [24.6–33.5]
BMI, kg/m^2^	23.4 [20.5–27.4]
BMI < 18.5 kg/m^2^, *n*/N (%)	16/211 (7.6%)
BMI 18.5–24.9 kg/m^2^, *n*/N (%)	121/211 (57.3%)
BMI 25.0–29.9 kg/m^2^, *n*/N (%)	36/211 (17.1%)
BMI ≥ 30.0 kg/m^2^, *n*/N (%)	38/211 (18.0%)
Waist circumference, cm	77.0 [68.0–93.0]
Age at menarche, years	13.0 [12.0–14.0]
Gravidity, *n*	0.0 [0.0–1.0]
Children, *n*	0.0 [0.0–0.0]
Irregular menstrual cycle	122/210 (58.1%)
Dysmenorrhea severity (1–5)	3.0 [3.0–4.0]
Self-reported hirsutism	100/205 (48.8%)
Acne	117/211 (55.5%)
Oily skin/seborrhea	142/212 (67.0%)
Infertility diagnosis	57/206 (27.7%)
Prior hormonal testing	164/209 (78.5%)
Diagnosed obesity	38/212 (17.9%)
Any chronic disease	54/212 (25.5%)
Reported anxiety	79/212 (37.3%)
Reported depression	25/212 (11.8%)
Reported fear of infertility	29/212 (13.7%)

Data are median [IQR] or *n*/N (%); denominators differ where data were missing (Table S1). BMI categories follow the World Health Organization classification. Age was missing for respondents with an implausible date of birth. Of the 212 respondents, 159 reported a previous diagnosis of polycystic ovary syndrome (the terminology in use when the survey was administered in 2023), 40 reported no previous diagnosis and 13 did not answer the diagnostic-status item. The same characteristics by self-reported diagnostic status are given in Table S2 (classifiable respondents, *n* = 199), and those of the principal-model population in Table S6 (*n* = 194). BMI, body mass index; IQR, interquartile range.

**Table 2:** Patient-reported health satisfaction and symptom burden in the primary analytical dataset (*n* = 212).

Item (Score 1–5)	Direction	Median [IQR]
Overall quality-of-life satisfaction	↑ better	4.0 [3.0–4.0]
Health satisfaction	↑ better	3.0 [3.0–4.0]
Negative feelings	↓ worse	3.0 [3.0–4.0]
Sexual satisfaction	↑ better	3.0 [3.0–4.0]
Weight-related frustration	↓ worse	3.0 [2.0–4.0]
General pain frequency	↓ worse	3.0 [2.0–4.0]
Pain interference (ADL)	↓ worse	2.0 [2.0–3.0]
Premenstrual burden	↓ worse	4.0 [3.0–4.0]
Importance of appearance	—	4.0 [4.0–5.0]

Data are median [IQR] on a five-point scale. All items were written for this survey; the first two are single-item ratings of satisfaction with quality of life and with health (Section 2.3). ↑ higher score = better; ↓ higher score = worse. Importance of appearance is an attitudinal item with no better-or-worse pole and is reported descriptively only. The same items stratified by self-reported diagnostic status are given in Table S3 (classifiable respondents, *n* = 199). ADL, activities of daily living; IQR, interquartile range.

## Data Availability

The de-identified analytical dataset and the analysis code are available from the corresponding author on reasonable request, subject to ethical and institutional restrictions. The original platform export is not shared because it contains potentially identifying information.

## Supplementary Materials

The following supporting information can be downloaded at https://www.mdpi.com/article/10.3390/healthcare14213635/s1, File S1: Wording of the study invitation and of the questionnaire items used in the analyses; Table S1: Valid numbers and missing values by variable in the primary analytical dataset (*n* = 212; the population described in Table 1 and Table 2) and by self-reported diagnostic status; Table S2: Sociodemographic, anthropometric and clinical characteristics by self-reported diagnostic status (classifiable respondents in the primary analytical dataset, *n* = 199); Table S3: Patient-reported health satisfaction and symptom burden by self-reported diagnostic status (classifiable respondents in the primary analytical dataset, *n* = 199); Table S4: Parallel proportional-odds ordinal logistic regression model for overall quality-of-life satisfaction (*n* = 194); Table S5: STROBE Statement—checklist of items for cross-sectional studies; Table S6: Characteristics of the principal-model population (*n* = 194); Table S7: Unadjusted proportional-odds estimates for health satisfaction (one covariate per model; *n* = 194); Table S8: Principal model, sensitivity analyses and robustness checks for health satisfaction: odds ratios (95% CI) and *p*-values; Table S9: Distribution of health-satisfaction responses in the primary analytical dataset and the principal-model population; Table S10: Response patterns for the multi-response psychological-condition item (“Do you suffer from any psychological condition?”); Table S11: Sociodemographic characteristics of the primary analytical dataset and the principal-model population; Table S12: Treatment reported by respondents reporting a previous diagnosis of PCOS; Table S13: Additional sensitivity and specification analyses for health satisfaction: odds ratios (95% CI).

## Abbreviations

The following abbreviations are used in this manuscript:

ADL	activities of daily living
AIC	Akaike information criterion
BMI	body mass index
CHERRIES	Checklist for Reporting Results of Internet E-Surveys
CI	confidence interval
GAD-7	Generalized Anxiety Disorder 7-item scale
HADS	Hospital Anxiety and Depression Scale
HRQoL	health-related quality of life
IQR	interquartile range
OR	odds ratio
PCOS	polycystic ovary syndrome
PCOSQ	Polycystic Ovary Syndrome Questionnaire
PCOSQOL	Polycystic Ovary Syndrome Quality of Life scale
PMOS	polyendocrine metabolic ovarian syndrome
STROBE	Strengthening the Reporting of Observational Studies in Epidemiology
WHO	World Health Organization
WHOQOL-BREF	World Health Organization Quality of Life Brief Version

## References

[1] Neven A.C.H., Forslund M., Ranasinha S., Sethi P., Dhungana R.R., Mousa A., Tay C.T., Teede H., Boyle J.A. (2026). Prevalence of polycystic ovary syndrome: A global and regional systematic review and meta-analysis. Hum. Reprod. Update.

[2] Teede H.J., Tay C.T., Laven J.J.E., Dokras A., Moran L.J., Piltonen T.T., Costello M.F., Boivin J., Redman L.M., Boyle J.A. (2023). Recommendations From the 2023 International Evidence-based Guideline for the Assessment and Management of Polycystic Ovary Syndrome. J. Clin. Endocrinol. Metab..

[3] Teede H.J., Bahri Khomami M., Morman R., Laven J.S.E., Joham A.E., Costello M.F., Patil M., Rees D.A., Berry L., Cree M.G. (2026). Polyendocrine metabolic ovarian syndrome, the new name for polycystic ovary syndrome: A multistep global consensus process. Lancet.

[4] Escobar-Morreale H.F. (2018). Polycystic ovary syndrome: Definition, aetiology, diagnosis and treatment. Nat. Rev. Endocrinol..

[5] Fahs D., Salloum D., Nasrallah M., Ghazeeri G. (2023). Polycystic Ovary Syndrome: Pathophysiology and Controversies in Diagnosis. Diagnostics.

[6] Otaghi M., Azami M., Khorshidi A., Borji M., Tardeh Z. (2019). The association between metabolic syndrome and polycystic ovary syndrome: A systematic review and meta-analysis. Diabetes Metab. Syndr..

[7] Cooney L.G., Lee I., Sammel M.D., Dokras A. (2017). High prevalence of moderate and severe depressive and anxiety symptoms in polycystic ovary syndrome: A systematic review and meta-analysis. Hum. Reprod..

[8] Li Y., Li Y., Ng E.H.Y., Stener-Victorin E., Hou L., Wu T., Han F., Wu X. (2011). Polycystic ovary syndrome is associated with negatively variable impacts on domains of health-related quality of life: Evidence from a meta-analysis. Fertil. Steril..

[9] Bazarganipour F., Taghavi S.A., Montazeri A., Ahmadi F., Chaman R., Khosravi A. (2015). The impact of polycystic ovary syndrome on the health-related quality of life: A systematic review and meta-analysis. Iran. J. Reprod. Med..

[10] Cronin L., Guyatt G., Griffith L., Wong E., Azziz R., Futterweit W., Cook D., Dunaif A. (1998). Development of a health-related quality-of-life questionnaire (PCOSQ) for women with polycystic ovary syndrome (PCOS). J. Clin. Endocrinol. Metab..

[11] Bazarganipour F., Ziaei S., Montazeri A., Faghihzadeh S., Frozanfard F. (2012). Psychometric properties of the Iranian version of modified polycystic ovary syndrome health-related quality-of-life questionnaire. Hum. Reprod..

[12] Rzońca E., Bień A., Wdowiak A., Szymański R., Iwanowicz-Palus G. (2018). Determinants of Quality of Life and Satisfaction with Life in Women with Polycystic Ovary Syndrome. Int. J. Environ. Res. Public Health.

[13] Hofmann K., Decrinis C., Bitterlich N., Tropschuh K., Stute P., Bachmann A. (2025). Body image and mental health in women with polycystic ovary syndrome–a cross-sectional study. Arch. Gynecol. Obstet..

[14] Jannink T.I., Bordewijk E.M., Lehmann V., Hoek A., Goddijn M., van Wely M. (2025). Patient characteristics associated with symptoms of anxiety, depression, and reduced body appreciation in women with polycystic ovary syndrome. Hum. Reprod..

[15] Ligocka N., Chmaj-Wierzchowska K., Wszołek K., Wilczak M., Tomczyk K. (2024). Quality of Life of Women with Polycystic Ovary Syndrome. Medicina.

[16] Stevanovic D., Bozic-Antic I., Stanojlovic O., Vojnovic Milutinovic D., Bjekic-Macut J., Jancic J., Macut D. (2019). Health-related quality of life questionnaire for polycystic ovary syndrome (PCOSQ-50): A psychometric study with the Serbian version. Women Health.

[17] Daescu A.-M.C., Dehelean L., Navolan D.-B., Gaitoane A.-I., Daescu A., Stoian D. (2023). Effects of Hormonal Profile, Weight, and Body Image on Sexual Function in Women with Polycystic Ovary Syndrome. Healthcare.

[18] von Elm E., Altman D.G., Egger M., Pocock S.J., Gøtzsche P.C., Vandenbroucke J.P., for the STROBE Initiative (2007). The Strengthening the Reporting of Observational Studies in Epidemiology (STROBE) statement: Guidelines for reporting observational studies. Lancet.

[19] Eysenbach G. (2004). Improving the quality of Web surveys: The Checklist for Reporting Results of Internet E-Surveys (CHERRIES). J. Med. Internet Res..

[20] Skevington S.M., Lotfy M., O’Connell K.A., WHOQOL Group (2004). The World Health Organization’s WHOQOL-BREF quality of life assessment: Psychometric properties and results of the international field trial. A report from the WHOQOL group. Qual. Life Res..

[21] Brant R. (1990). Assessing Proportionality in the Proportional Odds Model for Ordinal Logistic Regression. Biometrics.

[22] Amrhein V., Greenland S., McShane B. (2019). Scientists rise up against statistical significance. Nature.

[23] Bahri Khomami M., Ramezani Tehrani F., Hashemi S., Farahmand M., Azizi F. (2015). Of PCOS Symptoms, Hirsutism Has the Most Significant Impact on the Quality of Life of Iranian Women. PLoS ONE.

[24] Scragg J., Hobson A., Willis L., Taylor K.S., Dixon S., Jebb S.A. (2024). Effect of Weight Loss Interventions on the Symptomatic Burden and Biomarkers of Polycystic Ovary Syndrome: A Systematic Review of Randomized Controlled Trials. Ann. Intern. Med..

[25] Dokras A., Stener-Victorin E., Yildiz B.O., Li R., Ottey S., Shah D., Epperson N., Teede H. (2018). Androgen Excess–Polycystic Ovary Syndrome Society: Position statement on depression, anxiety, quality of life, and eating disorders in polycystic ovary syndrome. Fertil. Steril..

[26] Bahadori F., Jahanian Sadatmahalleh S., Mirzaei N. (2021). Health related quality of life and psychological parameters in different polycystic ovary syndrome phenotypes: A comparative cross-sectional study. J. Ovarian Res..

[27] DeSalvo K.B., Bloser N., Reynolds K., He J., Muntner P. (2006). Mortality prediction with a single general self-rated health question. A meta-analysis. J. Gen. Intern. Med..

[28] Jones G.L., Benes K., Clark T.L., Denham R., Holder M.G., Haynes T.J., Mulgrew N.C., Shepherd K.E., Wilkinson V.H., Singh M. (2004). The Polycystic Ovary Syndrome Health-Related Quality of Life Questionnaire (PCOSQ): A validation. Hum. Reprod..

[29] Williams S., Sheffield D., Knibb R.C. (2018). The Polycystic Ovary Syndrome Quality of Life scale (PCOSQOL): Development and preliminary validation. Health Psychol. Open.

[30] Pasch L., He S.Y., Huddleston H., Cedars M.I., Beshay A., Zane L.T., Shinkai K. (2016). Clinician vs self-ratings of hirsutism in patients with polycystic ovarian syndrome: Associations with quality of life and depression. JAMA Dermatol..

[31] Jiskoot G., van der Kooi A.-L., Busschbach J., Laven J., Beerthuizen A. (2022). Cognitive behavioural therapy for depression in women with PCOS: Systematic review and meta-analysis. Reprod. Biomed. Online.

[32] Ismayilova M., Yaya S. (2022). “I felt like she didn’t take me seriously”: A multi-methods study examining patient satisfaction and experiences with polycystic ovary syndrome (PCOS) in Canada. BMC Women’s Health.

[33] Mishra G.D., Hockey R., Powers J., Loxton D., Tooth L., Rowlands I., Byles J., Dobson A. (2014). Recruitment via the Internet and Social Networking Sites: The 1989–1995 Cohort of the Australian Longitudinal Study on Women’s Health. J. Med. Internet Res..

[34] The Rotterdam ESHRE/ASRM-sponsored PCOS consensus workshop group (2004). Revised 2003 consensus on diagnostic criteria and long-term health risks related to polycystic ovary syndrome (PCOS). Hum. Reprod..

[35] Lizneva D., Suturina L., Walker W., Brakta S., Gavrilova-Jordan L., Azziz R. (2016). Criteria, prevalence, and phenotypes of polycystic ovary syndrome. Fertil. Steril..

[36] Sterne J.A.C., White I.R., Carlin J.B., Spratt M., Royston P., Kenward M.G., Wood A.M., Carpenter J.R. (2009). Multiple imputation for missing data in epidemiological and clinical research: Potential and pitfalls. BMJ.

[37] Madley-Dowd P., Hughes R., Tilling K., Heron J. (2019). The proportion of missing data should not be used to guide decisions on multiple imputation. J. Clin. Epidemiol..

