# Supplementary material for: Correlates of Health Satisfaction in a Web-Based Sample Enriched for Self-Reported Polycystic Ovary Syndrome: An Exploratory Cross-Sectional Study
Source: Healthcare (Basel). 2026 Oct 27;14(21):3635. doi: 10.3390/healthcare14213635 (PMC13762933; doi:10.3390/healthcare14213635)
Supplement: Supplementary file 1 [file healthcare-14-03635-s001.zip › healthcare-4620517-supplementary.pdf]

Supplementary Materials

Correlates of Health Satisfaction in a Web-Based Sample Enriched for Self-Reported Polycystic Ovary Syndrome: An Exploratory Cross-Sectional Study

File S1. Wording of the study invitation and of the questionnaire items used in the analyses. Romanian item wording was reproduced from the original survey export; response options are those documented in the available source material. English translations were prepared by the authors. The same questionnaire was available online through a private Microsoft Forms link (Microsoft Corporation, Redmond, WA, USA) and, on request, as a PDF sent by e-mail; item wording and response options were identical in both versions. Of the 219 questionnaire records, 177 were completed directly online and 42 using the PDF version; the completed PDF questionnaires were entered into the same electronic questionnaire system by one author, and the transcribed responses were independently checked against the original PDF forms by a second author. Consent was acknowledged on an introductory page online and documented on the consent form for the PDF version. The invitation is reproduced below the table.

Variable	Item as exported (Romanian)	English translation	Response options documented	Coding
Health satisfaction (principal outcome)	În ce măsură sunteți mulțumită de starea dumneavoastră de sănătate?	To what extent are you satisfied with your state of health?	Deloc / În mică măsură / Nici în mare măsură, nici în mică măsură / În mare măsură / În foarte mare măsură (not at all / to a small extent / neither to a great nor to a small extent / to a great extent / to a very great extent)	Scored 1–5
Overall quality-of-life satisfaction	În ce măsură sunteți mulțumită de calitatea vieții dumneavoastră, in general?	To what extent are you satisfied with your quality of life, in general?	Deloc / În mică măsură / Nici în mare măsură, nici în mică măsură / În mare măsură / În foarte mare măsură	Scored 1–5
Frequency of negative feelings	Cât de frecvent aveți sentimente negative, cum ar fi stari de anxietate, depresie, disperare?	How often do you have negative feelings, such as anxiety, depression, despair?	Foarte rar / Rar / Uneori / Des / Foarte des (very rarely / rarely / sometimes / often / very often)	Scored 1–5
Sexual satisfaction	Cât de mulțumită sunteți de viața dvs sexuală?	How satisfied are you with your sex life?	Deloc / În mică măsură / Nici în mare măsură, nici în mică măsură / În mare măsură / În foarte mare măsură	Scored 1–5
Weight-related frustration	În ce măsură vă frustrează greutatea dvs?	To what extent does your weight frustrate you?	Deloc / În mică măsură / Nici în mare măsură, nici în mică măsură / În mare măsură / În foarte mare măsură	Scored 1–5
Frequency of physical pain	Cât de frecvent simțiți durere fizică, in general?	How often do you feel physical pain, in general?	Foarte rar / Rar / Uneori / Des / Foarte des	Scored 1–5
Pain interference	În ce măsură durerea sau disconfortul fizic vă împiedică să realizați activitățile zilnice?	To what extent does physical pain or discomfort prevent you from carrying out your daily activities?	Deloc / În mică măsură / Nici în mare măsură, nici în mică măsură / În mare măsură / În foarte mare măsură	Scored 1–5
Premenstrual symptom burden	În ce măsură vă este afectată starea fizică sau psihică înainte de ciclul menstrual? (sindrom premenstrual)	To what extent is your physical or mental state affected before your menstrual period? (premenstrual syndrome)	Deloc / În mică măsură / Nici în mare măsură, nici în mică măsură / În mare măsură / În foarte mare măsură	Scored 1–5
Importance of appearance	În ce măsură este important modul în care arătați din punct de vedere fizic pentru dvs?	To what extent is the way you look physically important to you?	Deloc / În mică măsură / Nici în mare măsură, nici în mică măsură / În mare măsură / În foarte mare măsură	Scored 1–5
Age	Data nașterii	Date of birth	Date	Age in years at 17 November 2023; implausible dates of birth (n = 5) treated as missing
Education	Studii	Education	Gimnaziale; Liceu; Studii superioare; Studii	1 = secondary or less (lower and

			postuniversitare (lower secondary; upper secondary; higher education; postgraduate)	upper secondary combined); 2 = higher education (bachelor level); 3 = postgraduate
Residential environment	Mediu	Residential environment	Urban; Rural	Rural = 1, urban = 0
Marital status	Starea civilă	Marital status	Căsătorită; Într-o relație; Singură; Divorțată (married; in a relationship; single; divorced)	1 = married; 2 = in a relationship; 3 = single or divorced
Occupation	Ocupația	Occupation	Free text	Coded into student or pupil; health professional; other employed or self-employed; not in paid employment; entries without an informative answer = missing
Height / weight	Ce înălțime aveți? (cm) / Ce greutate aveți? (kg)	What is your height? (cm) / What is your weight? (kg)	Free numeric entry	Metres converted to cm; weight > 200 or < 30 kg set to missing; BMI derived programmatically
Waist circumference	Ce circumferință abdominală aveți? (cm)	What is your abdominal circumference? (cm)	Free entry	Numeric component extracted; "do not know" and dashes missing
Age at menarche	Ce vârstă aveați când ați avut prima menstruație?	How old were you when you had your first menstruation?	Numeric	As reported
Gravidity	De câte ori ați fost însărcinată? (include număr sarcini duse la termen, avorturi spontane sau la cerere)	How many times have you been pregnant? (including pregnancies carried to term, spontaneous or induced abortions)	Free text	Coded numerically
Number of children	Câți copii aveți?	How many children do you have?	Free text	Coded numerically
Menstrual regularity	Aveți un ciclu menstrual:	Your menstrual cycle is:	Neregulat; Nu am ciclu menstrual; Regulat (mereu la același număr de zile) (irregular; I have no menstrual cycle; regular, always the same number of days)	Irregular = 1, regular = 0, no cycle = missing
Dysmenorrhea severity	Cum ați caracteriza durerile pe care le simțiți în timpul menstruației?	How would you describe the pain you feel during menstruation?	Foarte slabe / Slabe / Moderate / Puternice / Foarte puternice (very mild / mild / moderate / severe / very severe)	Scored 1–5
Self-reported hirsutism	Aveți hirsutism (pilozitate accentuată, păr în exces pe torace, față, fese)?	Do you have hirsutism (marked hairiness, excess hair on the chest, face, buttocks)?	Da; Nu; Nu știu/nu răspund (yes; no; do not know / no answer)	Yes = 1, no = 0, do not know / no answer = missing
Acne	Aveți acnee?	Do you have acne?	Da; Nu; Nu răspund	Yes = 1, no = 0, no answer = missing
Oily skin/seborrhea	Aveți tenul gras? (hiperseboree, ten lucios)	Do you have oily skin? (hyperseborrhoea, shiny complexion)	Da; Nu	Yes = 1, no = 0
Prior hormonal testing	Ați făcut vreodată dozări hormonale? (analize de sânge pentru hormoni)	Have you ever had hormone measurements? (blood tests for hormones)	Da; Nu; Nu știu	Yes = 1, no = 0, do not know = missing
Infertility diagnosis	Ați primit diagnostic de infertilitate?	Have you received a diagnosis of infertility?	Da; Nu; Nu răspund	Yes = 1, no = 0, no answer = missing
Chronic conditions (multi-response)	Cu ce afecțiuni cronice ați fost diagnosticată?	With which chronic conditions have you been diagnosed?	Nu am nicio afecțiune; Obezitate; Diabet zaharat tip 1 (tratat cu insulină); Diabet zaharat tip 2 (tratat cu pastile); Dislipidemie (trigliceride, colesterol crescut); Steatoză hepatică (ficat gras); Tromboză venoasă profundă	Obesity ticked = diagnosed obesity; any condition ticked = any chronic condition
Psychological condition (multi-response)	Suferiți de vreo afectare de ordin psihic?	Do you suffer from any psychological condition?	Anxietate; Depresie; Frica de infertilitate; Nu știu/nu răspund (no explicit "no" option)	Option selected = reporting the condition; not selected (including "do not know / no answer") = not

				reporting it
Self-reported diagnostic status	Dacă sunteți diagnosticată cu SOPC (sindromul ovarelor polichistice), ce anume v-a determinat să mergeți la medic?	If you have been diagnosed with PCOS (polycystic ovary syndrome), what prompted you to see a doctor?	ciclurile neregulate; hirsutismul; acneea; creșterea în greutate; infertilitatea; dureri puternice; flux menstrual abundent; altele; Nu sunt diagnosticată cu PCOS (irregular cycles; hirsutism; acne; weight gain; infertility; severe pain; heavy menstrual flow; other; "I am not diagnosed with PCOS")	Multiple selections permitted. "I am not diagnosed with PCOS" = no reported diagnosis; one or more reasons for seeking care = reported diagnosis; blank = undetermined. No response combined the two
Treatment	Dacă sunteți diagnosticată cu SOPC, urmați vreun tratament?	If you are diagnosed with PCOS, are you following any treatment?	Nu iau tratament; Contraceptive orale (anticoncepționale); Metformin; Myo Inozitol; Spironolactonă; Flutasin; Altceva (no treatment; oral contraceptives; metformin; myo-inositol; spironolactone; flutamide; other)	Each option coded 0/1; any treatment = any option other than "Nu iau tratament" (one response selecting both coded as treated). Conditional wording; answered by 158 of 159 respondents reporting a diagnosis and 18 of 40 reporting none in the primary analytical dataset. Summarised and modelled for respondents reporting a diagnosis (Tables S12 and S13); responses from respondents reporting none are described in the note to Table S12 and were not used to determine diagnostic status or included in the regression models

Other questionnaire items were not used in the analyses. Ethnicity, religion and the e-mail field of the export were not used and are not included in the analytical dataset; occupation is included only as a coded group.

Invitation circulated with the questionnaire (Romanian original)

Bună ziua!

Vă invităm să participați la un studiu medical dedicat **simptomatologiei sindromului ovarelor polichistice (PCOS)** și impactului acesteia asupra calității vieții.

Participarea presupune doar completarea unui scurt chestionar, prin care ne dorim să înțelegem mai bine experiențele pacientelor.

Participarea este complet voluntară, nu reprezintă o obligație, iar răspunsurile vor fi tratate confidențial și utilizate exclusiv în scopul cercetării.

Orice contribuție este binevenită și apreciată!

Vă mulțumim frumos pentru timpul acordat!

English translation

Good day!

We invite you to take part in a medical study dedicated to the **symptomatology of polycystic ovary syndrome (PCOS)** and its impact on quality of life.

Participation involves only completing a short questionnaire, through which we wish to understand patients' experiences better.

Participation is entirely voluntary and is not an obligation, and responses will be treated confidentially and used exclusively for research purposes.

Any contribution is welcome and appreciated!

Thank you very much for your time!

Table S1. Valid numbers and missing values by variable in the primary analytical dataset (n = 212; the population described in Tables 1 and 2) and by self-reported diagnostic status.

Variable	Valid n (of 212)	Missing, total	Missing: reported diagnosis (n = 159)	Missing: no diagnosis (n = 40)	Missing: undetermined (n = 13)
Age, years	207	5	4	0	1
BMI, kg/m²	211	1	0	0	1
Waist circumference, cm	161	51	38	9	4
Age at menarche, years	212	0	0	0	0
Gravidity	203	9	7	2	0
Number of children	205	7	5	2	0
Irregular menstrual cycle	210	2	2	0	0
Dysmenorrhea severity (1–5)	212	0	0	0	0
Self-reported hirsutism	205	7	4	2	1
Acne	211	1	1	0	0
Oily skin/seborrhea	212	0	0	0	0
Infertility diagnosis	206	6	5	1	0
Prior hormonal testing	209	3	2	1	0
Diagnosed obesity	212	0	0	0	0
Any chronic condition	212	0	0	0	0
Reported anxiety	212	0	0	0	0
Reported depression	212	0	0	0	0
Reported fear of infertility	212	0	0	0	0
Overall quality-of-life satisfaction	212	0	0	0	0
Health satisfaction	212	0	0	0	0
Frequency of negative feelings	212	0	0	0	0
Sexual satisfaction	212	0	0	0	0
Weight-related frustration	212	0	0	0	0
Frequency of physical pain	212	0	0	0	0
Pain interference with daily activities	212	0	0	0	0
Premenstrual symptom burden	212	0	0	0	0
Importance of physical appearance	212	0	0	0	0

The primary analytical dataset excludes the later submission of each of seven potential repeat-submission pairs identified by a post hoc integrity screen (identical date of birth, height and weight; Methods, Section 2.6). Missing values arise from "do not know / no answer" responses to single-choice items, blank or non-numeric free-text entries, the response "I have no menstrual cycle", implausible weight (> 200 or < 30 kg) and five implausible dates of birth (age treated as missing). Multi-response items have no missing values by construction. Of 212 submissions, 18 were excluded from the principal model (n = 194) because at least one covariate was missing: self-reported hirsutism 7, infertility diagnosis 6, age (implausible date of birth) 5, menstrual regularity 2, BMI 1; 2 lacked more than one covariate.

Table S2. Sociodemographic, anthropometric and clinical characteristics by self-reported diagnostic status (classifiable respondents in the primary analytical dataset, n = 199).

Characteristic	Reported diagnosis (n =	No reported diagnosis (n =	Effect estimate (95% CI)	p-value	Test

	159)	40)			
Age, years	28.1 [25.1–33.5]	27.0 [24.4–34.6]	0.40 (−1.60 to 2.20)	0.685	Mann–Whitney U
BMI, kg/m²	23.5 [20.8–28.0]	21.8 [20.1–25.1]	1.41 (−0.19 to 2.91)	0.084	Mann–Whitney U
Waist circumference, cm	80.0 [70.0–95.0]	70.0 [65.5–82.5]	6.00 (1.00 to 12.00)	0.015	Mann–Whitney U
Age at menarche, years	13.0 [12.0–14.0]	12.0 [12.0–13.2]	0 (0 to 1)	0.273	Mann–Whitney U
Gravidity	0.0 [0.0–1.0]	0.0 [0.0–0.8]	0 (0 to 0)	0.347	Mann–Whitney U
Number of children	0.0 [0.0–0.0]	0.0 [0.0–0.0]	0 (0 to 0)	0.914	Mann–Whitney U
Dysmenorrhea severity (1–5)	3.0 [3.0–4.0]	3.0 [3.0–4.0]	0 (0 to 0)	0.734	Mann–Whitney U
Irregular menstrual cycle	106/157 (67.5%)	13/40 (32.5%)	4.28 (1.95–9.85)	< 0.001	Fisher exact
Self-reported hirsutism	89/155 (57.4%)	11/38 (28.9%)	3.29 (1.46–7.90)	0.002	Fisher exact
Acne	95/158 (60.1%)	21/40 (52.5%)	1.36 (0.64–2.90)	0.473	Fisher exact
Oily skin/seborrhea	111/159 (69.8%)	25/40 (62.5%)	1.39 (0.62–3.02)	0.447	Fisher exact
Infertility diagnosis	52/154 (33.8%)	3/39 (7.7%)	6.07 (1.79–32.28)	< 0.001	Fisher exact
Prior hormonal testing	136/157 (86.6%)	22/39 (56.4%)	4.95 (2.11–11.69)	< 0.001	Fisher exact
Diagnosed obesity	33/159 (20.8%)	4/40 (10.0%)	2.35 (0.76–9.73)	0.171	Fisher exact
Any chronic condition	46/159 (28.9%)	7/40 (17.5%)	1.91 (0.76–5.50)	0.165	Fisher exact
Reported anxiety	61/159 (38.4%)	13/40 (32.5%)	1.29 (0.59–2.95)	0.584	Fisher exact
Reported depression	20/159 (12.6%)	3/40 (7.5%)	1.77 (0.49–9.80)	0.580	Fisher exact
Reported fear of infertility	26/159 (16.4%)	2/40 (5.0%)	3.70 (0.86–33.56)	0.076	Fisher exact

Data are median [IQR] or n/N (%). Effect estimates: for continuous and ordinal variables, the Hodges–Lehmann estimate of the location difference (reported diagnosis minus no reported diagnosis); for binary variables, the conditional maximum-likelihood odds ratio (reported versus no reported diagnosis) with exact 95% CI. Fisher's exact test was used for every binary comparison. Comparisons are exploratory and unadjusted for multiplicity; $p > 0.05$ does not indicate equivalence. Age was missing for respondents with an implausible date of birth.

Table S3. Patient-reported health satisfaction and symptom burden by self-reported diagnostic status (classifiable respondents in the primary analytical dataset, n = 199).

Item (score 1–5)	Reported diagnosis (n = 159)	No reported diagnosis (n = 40)	Location difference (95% CI)	p-value
Overall quality-of-life satisfaction ↑	4.0 [3.0–4.0]	4.0 [3.0–4.0]	0 (0 to 0)	0.752
Health satisfaction ↑	3.0 [3.0–4.0]	4.0 [3.0–4.0]	0 (−1 to 0)	0.089
Frequency of negative feelings ↓	3.0 [3.0–4.0]	3.0 [3.0–4.0]	0 (0 to 1)	0.544
Sexual satisfaction ↑	3.0 [3.0–4.0]	3.5 [3.0–4.0]	0 (0 to 0)	0.901
Weight-related frustration ↓	4.0 [2.0–4.0]	3.0 [2.0–4.0]	0 (0 to 1)	0.360
Frequency of physical pain ↓	3.0 [2.0–4.0]	3.0 [3.0–3.0]	0 (0 to 0)	0.849
Pain interference with daily activities ↓	2.0 [2.0–3.0]	2.0 [2.0–3.0]	0 (0 to 0)	0.871
Premenstrual symptom burden ↓	4.0 [3.0–4.0]	3.5 [2.0–4.0]	0 (0 to 1)	0.287
Importance of physical appearance	4.0 [4.0–5.0]	4.0 [4.0–4.0]	0 (0 to 0)	0.837

Data are median [IQR]. ↑ higher score = better; ↓ higher score = worse. Location difference: Hodges–Lehmann estimate (reported diagnosis minus no reported diagnosis) from the Mann–Whitney U test. No statistically detectable between-group difference was observed; this does not establish equivalence.

Table S4. Parallel proportional-odds ordinal logistic regression model for overall quality-of-life satisfaction (n = 194).

Variable	OR	95% CI	p-value
Age (per year)	0.99	0.95–1.04	0.800
BMI (per kg/m²)	0.93	0.89–0.99	0.013
Irregular cycle	0.98	0.55–1.75	0.942
Self-reported hirsutism	0.67	0.37–1.21	0.185
Infertility diagnosis	2.08	1.04–4.15	0.037
Dysmenorrhea (per level)	0.91	0.70–1.18	0.459
Reported anxiety	0.65	0.37–1.17	0.151

Same covariates and analysis population as the principal model (Table 3). OR < 1 indicates lower overall quality-of-life satisfaction. Likelihood-ratio $\chi^2 = 15.37$, df = 7, p = 0.032; McFadden pseudo-R^2 0.04; Nagelkerke pseudo-R^2 0.09; AIC 435.0. Brant omnibus $\chi^2 = 14.20$, df = 21, p = 0.86. The estimate for infertility diagnosis was nominally statistically significant but imprecise; it arose in a secondary exploratory model without adjustment for multiplicity and is not interpreted.

Table S5. STROBE Statement — checklist of items for cross-sectional studies

Correlates of Health Satisfaction in a Web-Based Sample Enriched for Self-Reported Polycystic Ovary Syndrome: An Exploratory Cross-Sectional Study

Section/Topic	Item	Recommendation (abridged)	Where reported
Title and abstract	1a	Indicate the study design with a commonly used term in the title or the abstract	Title; Abstract
	1b	Provide an informative and balanced summary of what was done and what was found	Abstract
Background/rationale	2	Explain the scientific background and rationale	Introduction
Objectives	3	State specific objectives, including any prespecified hypotheses	Introduction, final paragraph (no prespecified hypotheses; exploratory)
Study design	4	Present key elements of study design early in the paper	Section 2.1
Setting	5	Describe the setting, locations and relevant dates	Sections 2.1–2.2 (Romanian-language questionnaire administered using Microsoft Forms through a privately distributed link, with an identical PDF version available on request; 177 online and 42 PDF records; 10 October–17 November 2023)
Participants	6a	Eligibility criteria, sources and methods of selection	Section 2.2 (adult women; non-probability convenience sampling through patient contacts associated with medical practices and related groups)
Variables	7	Define all outcomes, exposures, predictors, potential confounders and effect modifiers	Sections 2.3–2.6; File S1 (item wording)
Data sources/measurement	8	Sources of data and methods of assessment	Sections 2.3–2.4 and 2.6; File S1 (study-specific, non-validated self-report items administered in Romanian; identical online

			and PDF versions, with PDF responses entered manually and checked independently by a second author; self-reported diagnostic status; no WHOQOL-BREF domain scores)
Bias	9	Describe any efforts to address potential sources of bias	Sections 2.1, 2.6, 2.7 and 4.6 (post hoc data-integrity screen; analysis retaining all submissions; selection bias acknowledged)
Study size	10	Explain how the study size was arrived at	Section 2.2 (all eligible respondents; no a priori sample-size calculation)
Quantitative variables	11	Explain how quantitative variables were handled	Sections 2.6–2.7 (age and BMI entered as continuous terms; quadratic age term, Table S8; quadratic and natural cubic spline terms for BMI and categorical dysmenorrhea severity, Table S13)
Statistical methods	12a	Describe all statistical methods, including those used to control for confounding	Section 2.7
	12b	Methods used to examine subgroups and interactions	Section 2.7 (restriction to reported diagnosis; no interaction terms)
	12c	Explain how missing data were addressed	Sections 2.6–2.7; Table S1 (complete-case analysis; no imputation; implausible dates of birth treated as missing)
	12d	If applicable, describe analytical methods taking account of sampling strategy	Not applicable (non-probability sample; no weighting)
	12e	Describe any sensitivity analyses	Section 2.7; Table S8 (three sensitivity analyses and two robustness checks); Table S13 (analyses defined after peer review: alternative coding of the psychological-condition item, sociodemographic and treatment adjustment, and specification of BMI, dysmenorrhea severity and the outcome categories)
Participants	13a	Report numbers of individuals at each stage of study	Section 3.1; Figure 1 (219 submissions → 212 in the primary analytical dataset → 194 in the principal complete-case model; 199 classifiable for the secondary diagnostic-status comparisons only; views and participation rate not available)
	13b	Give reasons for non-participation at each stage	Section 3.1; Figure 1; Table S1 (post hoc integrity screen: 7 later submissions; incomplete covariate data: 18; undetermined diagnostic status: 13, excluded from the secondary comparisons only)

	13c	Consider use of a flow diagram	Figure 1
Descriptive data	14a	Characteristics of study participants	Table 1 (primary analytical dataset, n = 212); Table S6 (principal-model population, n = 194); Table S2 (classifiable respondents, n = 199); Table S10 (responses to the psychological-condition item, n = 212 and n = 194); Table S11 (sociodemographic characteristics, n = 212 and n = 194); Table S12 (treatment among respondents reporting a diagnosis)
	14b	Number of participants with missing data for each variable	Table S1 (primary analytical dataset, n = 212); Tables S11 and S12 (sociodemographic and treatment items)
Outcome data	15	Report numbers of outcome events or summary measures	Table 2 (primary analytical dataset, n = 212); Table S3 (classifiable respondents, n = 199); Table S9 (distribution of health satisfaction, n = 212 and n = 194)
Main results	16a	Unadjusted and adjusted estimates with precision	Table 3 and Figure 2 (principal model, n = 194); Table S7 (unadjusted estimates)
	16b	Report category boundaries when continuous variables were categorised	Table 1 (World Health Organization BMI categories)
	16c	Translate estimates of relative risk into absolute risk if relevant	Not applicable (ordinal outcome)
Other analyses	17	Report other analyses done	Sections 3.3–3.6; Tables S2–S4 (secondary, exploratory), S8 (sensitivity and robustness analyses) and S13 (sensitivity and specification analyses defined after peer review); Tables S9–S12 (supporting descriptive data)
Key results	18	Summarise key results with reference to study objectives	Section 4.1
Limitations	19	Discuss limitations, including sources of bias or imprecision	Section 4.6
Interpretation	20	Give a cautious overall interpretation	Sections 4.3–4.4 and 5
Generalisability	21	Discuss the generalisability (external validity)	Sections 4.4 and 4.6 (predominantly young, urban and highly educated sample; no generalisation beyond similarly recruited samples)
Funding	22	Give the source of funding and the role of the funders	Funding statement (no external funding)

Adapted from the STROBE statement for cross-sectional studies (von Elm et al., reference 18 of the manuscript). Items that could not be fully reported are indicated in the last column.

Table S6. Characteristics of the principal-model population (n = 194).

Characteristic	Value
Reported diagnosis	145/194 (74.7%)
No reported diagnosis	37/194 (19.1%)
Undetermined status	12/194 (6.2%)
Age, years	28.4 [24.6–33.7] (n = 194)
BMI, kg/m²	23.4 [20.4–27.4] (n = 194)
Waist circumference, cm	76.5 [67.8–90.0] (n = 148)
Irregular menstrual cycle	112/194 (57.7%)
Dysmenorrhea severity (1-5)	3.0 [3.0–4.0] (n = 194)
Self-reported hirsutism	97/194 (50.0%)
Acne	104/194 (53.6%)
Oily skin/seborrhea	129/194 (66.5%)
Infertility diagnosis	53/194 (27.3%)
Prior hormonal testing	150/191 (78.5%)
Diagnosed obesity	34/194 (17.5%)
Reported anxiety	69/194 (35.6%)
Reported depression	23/194 (11.9%)

Data are median [IQR] (n with data) or n/N (%). The principal model included all respondents in the primary analytical dataset with complete covariate data, irrespective of diagnostic status.

Table S7. Unadjusted proportional-odds estimates for health satisfaction (one covariate per model; n = 194).

Variable	OR	95% CI	p-value
Age (per year)	0.98	0.94–1.03	0.480
BMI (per kg/m²)	0.90	0.86–0.95	< 0.001
Irregular cycle	0.73	0.43–1.24	0.247
Self-reported hirsutism	0.35	0.20–0.60	< 0.001
Infertility diagnosis	0.69	0.38–1.22	0.202
Dysmenorrhea (per level)	0.86	0.67–1.10	0.215
Reported anxiety	0.42	0.24–0.73	0.002

Each model contains a single covariate and is fitted on the principal analysis population. Adjusted estimates are given in Table 3.

Table S8. Principal model, sensitivity analyses and robustness checks for health satisfaction: odds ratios (95% CI) and p-values.

Variable	A. Principal model (n = 194)	B. Diagnosis-restricted (n = 145)	C. Diagnostic-status-adjusted (n = 182)	D. All submissions (n = 200)	E. Quadratic age (n = 194)	F. High-similarity check (n = 193)
Age (per year)	0.98 (0.93–1.03); p = 0.403	0.98 (0.92–1.04); p = 0.465	0.98 (0.93–1.03); p = 0.416	0.99 (0.94–1.04); p = 0.606	—	0.98 (0.93–1.03); p = 0.397
Age, mean-centred (per year)	—	—	—	—	1.01 (0.95–1.07); p = 0.768	—
Age, mean-centred, squared	—	—	—	—	0.99 (0.99–1.00); p = 0.054	—
BMI (per kg/m²)	0.92 (0.87–0.97); p = 0.001	0.93 (0.88–0.98); p = 0.011	0.91 (0.86–0.96); p < 0.001	0.91 (0.87–0.96); p < 0.001	0.92 (0.87–0.96); p < 0.001	0.91 (0.86–0.96); p < 0.001
Irregular cycle	1.05 (0.60–1.84); p = 0.876	1.13 (0.58–2.20); p = 0.715	1.16 (0.63–2.10); p = 0.636	1.07 (0.61–1.87); p = 0.815	1.07 (0.61–1.88); p = 0.822	1.02 (0.58–1.79); p = 0.956

Self-reported hirsutism	0.43 (0.24–0.76); p = 0.004	0.55 (0.29–1.05); p = 0.069	0.45 (0.25–0.82); p = 0.009	0.42 (0.24–0.75); p = 0.003	0.42 (0.24–0.76); p = 0.004	0.42 (0.23–0.75); p = 0.003
Infertility diagnosis	1.08 (0.56–2.08); p = 0.815	0.97 (0.48–1.94); p = 0.920	1.11 (0.57–2.17); p = 0.760	0.93 (0.49–1.78); p = 0.837	0.91 (0.46–1.81); p = 0.795	1.08 (0.56–2.07); p = 0.829
Dysmenorrhea (per level)	0.88 (0.67–1.14); p = 0.335	0.73 (0.54–0.99); p = 0.042	0.87 (0.66–1.14); p = 0.300	0.95 (0.73–1.23); p = 0.706	0.90 (0.69–1.18); p = 0.462	0.86 (0.66–1.12); p = 0.264
Reported anxiety	0.49 (0.28–0.87); p = 0.014	0.68 (0.35–1.31); p = 0.248	0.53 (0.30–0.96); p = 0.035	0.46 (0.26–0.81); p = 0.007	0.50 (0.28–0.89); p = 0.019	0.48 (0.27–0.85); p = 0.012
No reported diagnosis (vs reported diagnosis)	—	—	1.00 (0.48–2.06); p = 0.993	—	—	—
Nagelkerke pseudo-R^2	0.18	0.15	0.17	0.19	0.20	0.20
Brant omnibus test, p	0.44	0.48	0.49	0.57	0.46	0.43
Smallest covariate-specific Brant p	0.20	0.07	0.08	0.16	0.20	0.18

All models are proportional-odds ordinal logistic regressions for health satisfaction; OR < 1 indicates lower health satisfaction. A: primary analytical dataset (later submission of each of seven potential repeat-submission pairs excluded). B: respondents reporting a diagnosis of PCOS. C: classifiable respondents, with diagnostic status added as a covariate; its estimate is imprecise and is not interpreted as evidence about the effect of diagnosis. D: all submissions, including the seven later submissions. E: linear age replaced by mean-centred age and its square (comparison with the linear model: likelihood-ratio $\chi^2 = 3.74$, df = 1, p = 0.053). F: principal model after excluding the later of one additional pair of highly similar submissions (33 of 44 fields identical) that did not meet the matching rule; these submissions are not treated as duplicates. Hirsutism was self-reported; anxiety was reported on a multi-response item without an explicit "no" option. These analyses were not prespecified and are exploratory.

Table S9. Distribution of health-satisfaction responses in the primary analytical dataset and the principal-model population.

**Response category (score)**	**Primary analytical dataset (n = 212), n (%)**	**Principal-model population (n = 194), n (%)**
Not at all (1)	9 (4.2%)	7 (3.6%)
To a small extent (2)	38 (17.9%)	33 (17.0%)
Neither to a great nor to a small extent (3)	63 (29.7%)	60 (30.9%)
To a great extent (4)	91 (42.9%)	84 (43.3%)
To a very great extent (5)	11 (5.2%)	10 (5.2%)

Item: "To what extent are you satisfied with your state of health?" (File S1); the item wording did not specify a reference period. Categories 1 and 5 were the least frequent; a model combining categories 1 and 2 is reported in Table S13.

Table S10. Response patterns for the multi-response psychological-condition item ("Do you suffer from any psychological condition?").

**Response pattern**	**Primary analytical dataset (n = 212), n (%)**	**Principal-model population (n = 194), n (%)**	**Coding in the principal model**
Anxiety only	49 (23.1%)	44 (22.7%)	Reported anxiety
Anxiety with depression and/or fear of infertility	30 (14.2%)	25 (12.9%)	Reported anxiety
Depression and/or fear of infertility, without anxiety	17 (8.0%)	15 (7.7%)	Not reporting anxiety (comparator)
"Do not know / no answer" only	116 (54.7%)	110 (56.7%)	Not reporting anxiety (comparator)
No option selected	0 (0.0%)	0 (0.0%)	–

The item offered anxiety, depression, fear of infertility and "do not know / no answer", without an explicit "no" option. "Do not know / no answer" was selected together with another option in 0 of the 219 submissions, and 0 respondents left the item blank. When "do not know / no answer" was treated as missing (Table S13), 84 respondents remained in the principal-model population, and the comparator consisted of the 15 respondents selecting depression and/or fear of infertility without anxiety.

Table S11. Sociodemographic characteristics of the primary analytical dataset and the principal-model population.

Characteristic	Primary analytical dataset (n = 212)	Principal-model population (n = 194)
Education		
Secondary education or less	24/212 (11.3%)	23/194 (11.9%)
Higher education (bachelor level)	139/212 (65.6%)	125/194 (64.4%)
Postgraduate education	49/212 (23.1%)	46/194 (23.7%)
Residential environment		
Urban	179/212 (84.4%)	166/194 (85.6%)
Rural	33/212 (15.6%)	28/194 (14.4%)
Marital status		
Married	93/212 (43.9%)	87/194 (44.8%)
In a relationship	84/212 (39.6%)	74/194 (38.1%)
Single or divorced	35/212 (16.5%)	33/194 (17.0%)
Occupation group		
Student or pupil	51/208 (24.5%)	46/191 (24.1%)
Health professional	23/208 (11.1%)	23/191 (12.0%)
Other employed or self-employed	128/208 (61.5%)	117/191 (61.3%)
Not in paid employment	6/208 (2.9%)	5/191 (2.6%)

Data are n/N (%). Education, residential environment and marital status were single-choice items answered in all submissions; lower secondary education (n = 2) was combined with upper secondary education and divorced (n = 3) with single. Occupation was recorded as free text and coded into four groups; entries without an informative answer were not classified (4 in the primary analytical dataset, 3 in the principal-model population).

Table S12. Treatment reported by respondents reporting a previous diagnosis of PCOS.

Treatment	Reporting a diagnosis, primary analytical dataset (n = 159)	Reporting a diagnosis, principal-model population (n = 145)
Answered the treatment item	158/159 (99.4%)	144/145 (99.3%)
No treatment	86/158 (54.4%)	78/144 (54.2%)
Any treatment	72/158 (45.6%)	66/144 (45.8%)
Any listed medicine (oral contraceptive, metformin, spironolactone or flutamide)	49/158 (31.0%)	45/144 (31.3%)
Myo-inositol only	21/158 (13.3%)	20/144 (13.9%)
Other, unspecified only	2/158 (1.3%)	1/144 (0.7%)
Individual options (multi-response)		
Oral contraceptive	28/158 (17.7%)	25/144 (17.4%)
Metformin	23/158 (14.6%)	22/144 (15.3%)
Myo-inositol (any)	34/158 (21.5%)	32/144 (22.2%)
Spironolactone	6/158 (3.8%)	6/144 (4.2%)
Flutamide	2/158 (1.3%)	1/144 (0.7%)
Other (unspecified)	2/158 (1.3%)	1/144 (0.7%)

Item: "If you are diagnosed with PCOS, are you following any treatment?" (multi-response; File S1). Percentages use respondents answering the item; one respondent may count under several individual options. One response selected both "no treatment" and two treatments; it was coded as treated, and alternative coding is examined in Table S13. The item was also

answered by 18 of the 40 respondents reporting no diagnosis (17 selecting no treatment); these responses were not used to determine diagnostic status or included in the treatment-adjusted regression models (Table S13).

Table S13. Additional sensitivity and specification analyses for health satisfaction: odds ratios (95% CI).

**Analysis (population)**	**BMI (per kg/m²)**	**Self-reported hirsutism**	**Reported anxiety**	**Additional terms or model comparison**
Principal model (n = 194)	0.92 (0.87–0.97)	0.43 (0.24–0.76)	0.49 (0.28–0.87)	–
Coding of the psychological-condition item				
"Do not know / no answer" treated as missing (n = 84)[d]	0.86 (0.80–0.93)	0.91 (0.37–2.26)	0.68 (0.22–2.11)	Comparator: respondents selecting depression and/or fear of infertility without anxiety
Three-category coding (n = 194)	0.92 (0.87–0.97)	0.42 (0.24–0.76)	0.47 (0.26–0.84)	Reference: "do not know / no answer" only; depression and/or fear of infertility without anxiety: 0.66 (0.22–1.93)
Sociodemographic adjustment				
Education and residential environment added (n = 194)	0.92 (0.87–0.97)	0.46 (0.26–0.84)	0.48 (0.27–0.85)	LR $\chi^2 = 6.35$, df = 3, $p = 0.096$
Education, residential environment and marital status added (n = 194)	0.92 (0.87–0.97)	0.47 (0.26–0.84)	0.48 (0.27–0.85)	LR $\chi^2 = 6.77$, df = 5, $p = 0.239$
Principal covariates, classifiable occupation (n = 191)	0.92 (0.87–0.97)	0.38 (0.21–0.69)	0.47 (0.26–0.84)	–
Occupation added (n = 191)[c,d]	0.92 (0.87–0.97)	0.38 (0.21–0.70)	0.47 (0.26–0.85)	LR $\chi^2 = 0.15$, df = 2, $p = 0.926$
Treatment (respondents reporting a diagnosis who answered the treatment item)				
Principal covariates (n = 144)	0.93 (0.88–0.98)	0.54 (0.28–1.04)	0.69 (0.36–1.33)	–
Any treatment added (n = 144)	0.93 (0.88–0.98)	0.53 (0.28–1.03)	0.68 (0.35–1.33)	Any treatment: 1.08 (0.57–2.04); LR $\chi^2 = 0.06$, df = 1, $p = 0.807$
As above, conflicting response coded untreated (n = 144)	0.93 (0.88–0.98)	0.54 (0.28–1.04)	0.69 (0.35–1.35)	Any treatment: 1.01 (0.53–1.91)
As above, conflicting response excluded (n = 143)	0.93 (0.88–0.98)	0.52 (0.27–1.01)	0.70 (0.36–1.38)	Any treatment: 1.03 (0.54–1.95)
Any listed medicine added (n = 144)	0.93 (0.88–0.98)	0.54 (0.28–1.03)	0.68 (0.35–1.33)	Any listed medicine: 1.07 (0.54–2.09); LR $\chi^2 = 0.03$, df = 1, $p = 0.853$
Model specification				
Quadratic BMI term (n = 194)	0.88 (0.82–0.95)[a]	0.43 (0.24–0.77)	0.48 (0.27–0.85)	Quadratic vs linear BMI: LR $\chi^2 = 2.54$, df = 1, $p = 0.111$
Natural cubic spline for BMI, 3 df (n = 194)[d]	–[b]	0.43 (0.24–0.77)	0.49 (0.27–0.87)	Spline vs linear BMI: LR $\chi^2 = 3.11$, df = 2, $p = 0.212$
Dysmenorrhea severity as categories (n = 194)[d]	0.91 (0.86–0.96)	0.43 (0.24–0.78)	0.48 (0.27–0.86)	Categories vs per-level coding: LR $\chi^2 = 2.71$, df = 3, $p = 0.438$; vs moderate: very mild 1.25 (0.43–3.68), mild 1.10 (0.42–2.87), severe 1.19 (0.62–2.31), very severe 0.50 (0.20–1.25)
Health-satisfaction categories 1 and 2 combined (n = 194)	0.91 (0.86–0.96)	0.43 (0.24–0.77)	0.47 (0.27–0.84)	–

All models are proportional-odds ordinal logistic regressions that retain the seven principal covariates (Table 3); they were defined after peer review, are exploratory and are not adjusted for multiplicity. OR < 1 indicates lower health satisfaction. Comparisons of treatment models are made within the same respondents. LR, likelihood-ratio test against the corresponding model without the added terms. [a] Per kg/m² at the mean BMI (BMI mean-centred). [b] Spline terms are not summarised by a single odds ratio. [c] Student or pupil and not

in paid employment, each compared with employed or self-employed respondents; health professionals were combined with other employed respondents because several free-text entries could not be assigned unambiguously to either group. [d] The proportional-odds assumption could not be assessed with the Brant test (see below). Numbers of respondents in the principal-model population by dysmenorrhea severity: very mild 18; mild 19; moderate 72; severe 62; very severe 23. All models converged with finite standard errors. The Brant test was evaluable in 11 of the 15 additional models, with omnibus p-values of 0.21–0.68. It was not evaluable, and no statistic is reported, where a covariate showed separation in one of the binary logistic submodels on which the test is based: in the model treating "do not know / no answer" as missing (the submodels also failed to converge), in the occupation model (no student or pupil and no respondent not in paid employment selected category 1) and in the categorical dysmenorrhea model (no respondent with mild or severe dysmenorrhea selected category 1, and none with very severe dysmenorrhea selected category 5). It could not be computed for the spline model.
